# Chemical profiling and evaluation of in vitro antibacterial and antibiotic synergistic activities of *Cleistanthus bracteosus* Jabl. from Malaysian rainforests

**DOI:** 10.1186/s12906-026-05439-7

**Published:** 2026-06-20

**Authors:** Mackingsley Kushan Dassanayake, Teng-Jin Khoo, Chien Hwa Chong, Christophe Wiart, Omar Ashraf Elfar, Rachael Symonds, Mohammed Tahir Ansari

**Affiliations:** 1 School of Pharmacy, Faculty of Science and Engineering, University of Nottingham Malaysia, Jalan Broga, Semenyih, 43500 Malaysia; 2 Department of Chemical and Environmental Engineering, Faculty of Science and Engineering, University of Nottingham Malaysia, Jalan Broga, Semenyih, 43500 Malaysia; 3 Institute of Tropical Biology and Conservation, University Malaysia Sabah, Jalan Sulaman, Kota Kinabalu, Sabah 88400 Malaysia; 4 School of Biological and Environmental Sciences, Faculty of Science, Liverpool John Moores University, James Parsons Building, Byrom St, Liverpool, L3 3AF UK; 5 Department of Pharmaceutics and Industrial Pharmacy, Faculty of Pharmacy, Cairo University, Kasr El-Aini, Cairo, 11562 Egypt; 6 Leicester School of Pharmacy, Faculty of Health and Life Sciences, De Montfort University, The Gateway, Leicester, LE1 9BH UK

**Keywords:** Antibacterial activity, Synergistic activity, Cleistanthus bracteosus, GIIs, FE-SEM, GC–MS, HPLC

## Abstract

**Background:**

Plants are rich and valuable sources of phytochemical constituents that can contribute to a variety of biological activities. The research in discovering novel antimicrobial compounds from plants has been of increased interest due to the global rise of antibiotic resistance. The present study aims to evaluate the in vitro antibacterial, antibiotic synergistic potential and the chemical profile of plant extracts associated with *Cleistanthus bracteosus* Jabl*.* found in Malaysian rainforests for the first time.

**Methods:**

The antibacterial activity and synergistic antibacterial activity of chloroform, methanol and *n*-hexane crude extracts of *C. bracteosus* were determined against bacterial pathogens of *Escherichia coli*, *Bacillus subtilis*, *Staphylococcus aureus, B*. *cereus*, *Pseudomonas aeruginosa*, *Salmonella typhi* and *Acinetobacter baumannii*. The chloroform extracts were subjected to fractionation, chemical profiling, and field emission scanning electron microscopic (FE-SEM) analysis.

**Results:**

The chloroform stem extract exhibited the highest antibacterial activity which indicated a mean inhibition zone diameter (IZD) of 10 ± 0.26 mm against the Gram-positive bacterium *B*. cereus. The best synergistic activity was shown for chloroform wood extract when combined with ampicillin against *A. baumannii,* with a growth inhibitory index (GIIs) of 1.33. Potassium clavulanate actively synergised with chloroform stem extract against *E. coli* ATCC 10536 (IZD = 27 ± 0.56 mm) and chloroform bark extract against *S. typhi* (IZD = 28 ± 0.54 mm). *C. bracteosus* extracts showed the presence of secondary metabolites like flavonoids, phenols, tannins, alkaloids, coumarins, saponins and traces of terpenoids, with chloroform extracts of bark, stem and wood consisting of 20 bioactive phytochemical compounds according to gas chromatography–mass spectrometry analysis (GC–MS). Further analysis with high-performance liquid chromatography (HPLC) implied the presence of digoxigenin and lauric acid. The FE-SEM observations further elucidated the effects of *C. bracteosus* chloroform extracts on the morphology of susceptible organisms.

**Conclusion:**

The findings of this investigation justify that chloroform extracts of *C. bracteosus* can function as a novel antibacterial agent with synergistic potential.

**Supplementary Information:**

The online version contains supplementary material available at https://doi.org/10.1186/s12906-026-05439-7.

## Background

For thousands of years, plants have played an important role in traditional systems of medicine and are constantly providing humankind with novel remedies. Traditional or folklore medicine refers to health care practices based on any ancient and cultural background, which differs from modern medicine [1]. According to the World Health Organisation (WHO), about 65–80% of the populations residing in the developing world rely on traditional medicine, which involves the harnessing of remedies from plants of medicinal value for their primary health care needs [2]. The research in discovering new and innovative antimicrobial compounds from plants has received increased attention due to the global outbreak of diseases caused by pathogens that show multiple resistance to synthetic antimicrobial agents [3].

Antibiotic resistance is a major issue affecting global public health. The focus on the significance of medicinal plants and traditional therapeutic systems in solving global primary health care has grown throughout recent years. However, the complex nature of issues related to antibiotic resistance remains unresolved. Over time, humans have relied on the usage of traditionally prepared herbal remedies for the treatment of various ailments [4]. These traditional therapeutic methods have been considered as possibly the safest alternative sources of antimicrobial agents available [5]. Many discoveries have highlighted that naturally existing compounds from plants are reservoirs of chemical agents with therapeutic properties [6]. Plants contain several unique properties that are beneficial to mankind, which has motivated the initiation of screening and testing their efficacy in order to exploit their antibacterial potential [7]. Hence, investigations based on the understanding of their properties, safety and efficacy have widened, where efforts have been taken to identify safe phytochemical compounds, and economical methods for controlling diseases. Interestingly, these naturally existing bioactive compounds from plants serve as a novel source for the management of infectious diseases caused by pathogenic microorganisms as an alternative to synthetic drugs and several phytochemical compounds have been extracted from whole plants or different parts of plants like leaves, bark, stem, roots, fruits, fruit rind, seeds and flowers [8].

*Cleistanthus bracteosus* Jabl. is a tropical plant found in Malaysian rainforests which belongs to the “*Phyllanthaceae*” family [9]. The similar findings of recent investigations indicate that crude extracts of petroleum ether, chloroform and acetone leaves and stem of *C. collinus* and aqueous and acetone leaves of *Cleistanthus patulus* have antibacterial activity against both Gram-positive and Gram-negative bacteria [10, 11]. Also, certain parts of *C. collinus* and *C*. *patulus* like fruits, leaves and roots have been used to treat acute gastrointestinal disorders and infections in folklore medicine [11, 12]. Similar species like *C. collinus* consist of glucosides like cleistanthin A, B, and other secondary metabolites such as diphyllin, which has the anti-fungal and anti-proliferative potential of cancerous cells of the human nasopharynx via DNA synthesis inhibition. [12]. The present study seeks to explore and assess, for the first time, the antibacterial and synergistic properties of crude extracts and isolated extract fractions derived from *C. bracteosus*. Furthermore, the results of this study will provide scientific evidence supporting the clinical application of this plant and can serve as a starting point for novel drug discovery from natural products. To the best of our cognizance, there are no systematic studies on traditional uses, chemical analysis, antibacterial activities or any other biological activities of *Cleistanthus bracteosus* Jabl.

## Materials and methods

### Collection and identification of plant material

**Fig. 1:**
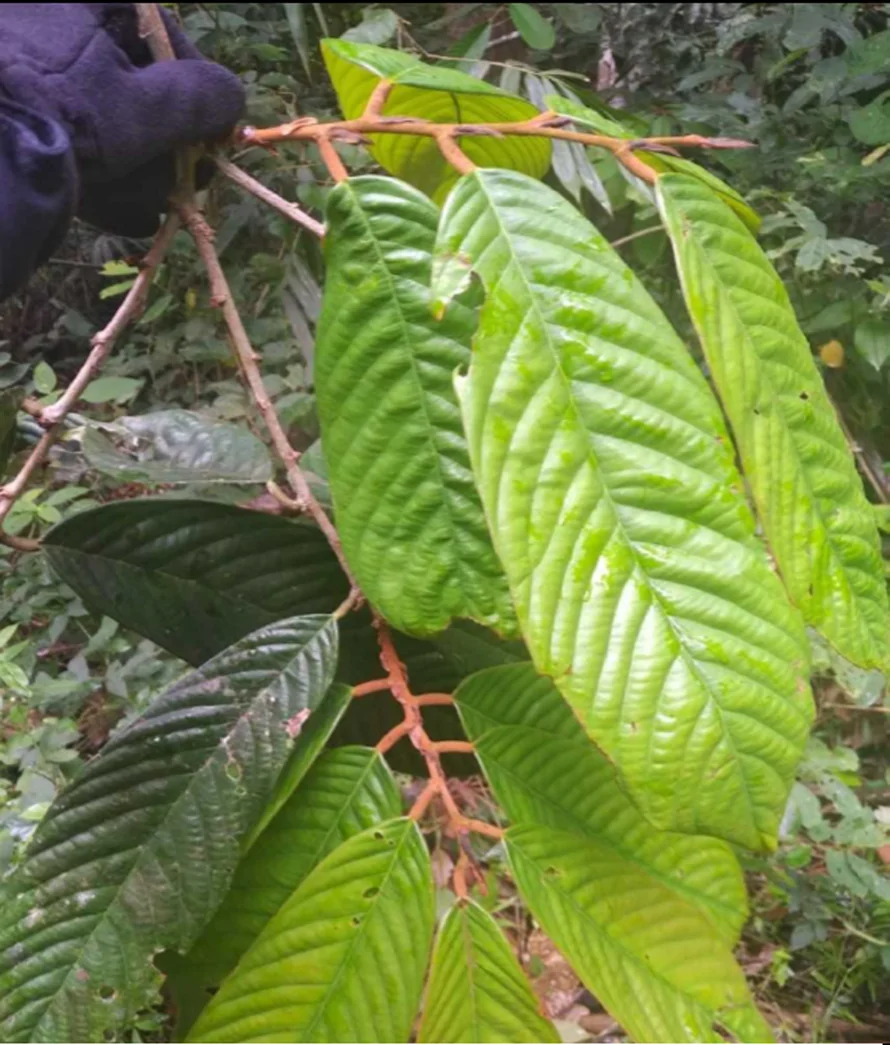
*C. bracteosus* in its natural habitat in Malaysia

Parts of the plant that include leaves, bark, stem and wood of *C*. *bracteosus* were collected from Manong primary rain forest in Perak, Peninsular Malaysia (GPS coordinates: 4°72″N 100°81″E) on 3rd February 2017 (Fig. 1). A sample of the whole plant was sent to the Forest Research Institute of Malaysia (FRIM) located in Kepong, Selangor for authentication. The plant was identified by Prof. Engkik Soepadmo at FRIM. A publicly made available herbarium voucher sample (NB125) of the whole authenticated plant of *C. bracteosus* was deposited under FRIM.

### Preparation of plant extracts

Leaves, bark, stem and wood of *C. bracteosus* were cleaned, air-dried and fragmented into pieces using the rotor beater mill (Retsch, Düsseldorf, Germany) and followed by the ultra-centrifugal mill (Retsch, Düsseldorf, Germany). The fragmented pieces were pulverized to a coarse powder with the application of a porcelain mortar and pestle. Seventy grams (70.0 g) of the powdered leaves, bark, stem and wood were separately dissolved in 1L of chloroform, methanol and *n*-hexane (Sigma-Aldrich, St. Louis, USA). Each mixture containing powdered plant material to solvent ratio of 1:14 (w/v) was macerated under a water bath at 40 °C for seven days and placed under an orbital shaker for another two days. Subsequently, the maceration was further assisted by ultrasonication at 40 °C for 30 min. The macerated mixture was filtered using Whatman grade 1 filter paper (GE Healthcare, Chicago, USA), and the solvent content of the macerated mixture was removed using the rotary evaporator under vacuum at 40 °C. Fifty milligrams (50) mg of the dried extract was reconstituted with 1 mL of pure DMSO (Sisco Research Laboratories, Mumbai, India), and the concentration of the stock solution was at 50 mg/mL.

### Yield calculation of extracts

The yield of plant extracted was calculated according to the following formula [13]:

$$\mathrm{Y}\mathrm{i}\mathrm{e}\mathrm{l}\mathrm{d} \left(\mathrm{\%}\right)= \frac{\mathrm{M}\mathrm{a}\mathrm{s}\mathrm{s}\,\mathrm{o}\mathrm{f}\,\mathrm{p}\mathrm{l}\mathrm{a}\mathrm{n}\mathrm{t}\,\mathrm{e}\mathrm{x}\mathrm{t}\mathrm{r}\mathrm{a}\mathrm{c}\mathrm{t}\mathrm{e}\mathrm{d}\,}{\mathrm{M}\mathrm{a}\mathrm{s}\mathrm{s}\,\mathrm{o}\mathrm{f}\,\mathrm{i}\mathrm{n}\mathrm{i}\mathrm{t}\mathrm{i}\mathrm{a}\mathrm{l}\,\mathrm{p}\mathrm{l}\mathrm{a}\mathrm{n}\mathrm{t}\,\mathrm{m}\mathrm{a}\mathrm{t}\mathrm{e}\mathrm{r}\mathrm{i}\mathrm{a}\mathrm{l}}\mathrm{x}\,100 \mathrm{\%}$$

### Preparation of bacterial isolates

Gram-positive bacteria *Bacillus subtilis* (ATCC 6633), *Staphylococcus aureus* (ATCC 11632) and *Bacillus cereus* (ATCC 10876) and Gram-negative bacteria *Escherichia coli* (ATCC 10536), *Escherichia coli* (ATCC 8739), *Pseudomonas aeruginosa* (ATCC 10145) and *Salmonella typhi* (ATCC 6539) were collected from the Microbiology laboratory, University of Nottingham Malaysia. *Acinetobacter baumannii* (ATCC 19606) was procured from SPD Scientific (M) SDN BHD. The OD UV absorbance value was monitored to be in the range of 0.08 to 0.10 at 625 nm in order to be adjusted to 0.5 McFarland turbidity standards which correspond to a bacterial cell count of 1.5 X 10^8^ CFU/mL [14].

### Assay for screening of antibacterial and antibiotic synergistic activity

The in vitro agar paper disk diffusion assay was used to evaluate the antibacterial activities of chloroform, methanol and *n*-hexane plant extracts. The antibacterial assay was performed on Muller-Hinton agar (Oxoid, Hampshire, UK). A sterile cotton swab was immersed in a bacterial suspension of 0.5 McFarland turbidity standards for each test organism and swabbed over the entire agar surface for even distribution. All extracts were brought to room temperature prior to being used in the assay. An aliquot equivalent to 20 μL of the reconstituted extract from the stock solution is transferred to sterilised filter paper disks (Whatman Grade 3; GE Healthcare, Chicago, USA) of 6 mm in diameter. The paper disks impregnated with pure DMSO was considered as the negative control and gentamicin 10 μg standard antibiotic disk was served as the positive control in standalone and antibiotic synergistic assays.

Standard antibiotic disks of gentamicin 10 μg, ciprofloxacin 5 μg, levofloxacin 5 μg, penicillin G 10 IU (6 µg), chloramphenicol 30 μg and ampicillin 10 μg (Oxoid, Hampshire, UK) were loaded and impregnated with 20 μL of the reconstituted plant extract for antibiotic synergistic activity testing [15]. Another set of plant extract disks were impregnated with 50 mg of potassium clavulanate (1:1 v/v). The impregnated disks were allowed to dry for few hours and set to a final concentration of 1000 μg/disk**.** The paper disks were placed on the agar plate using sterilized forceps. The paper disks impregnated with pure DMSO was considered as the negative control and gentamicin 10 μg, ciprofloxacin 5 μg, levofloxacin 5 μg, penicillin G 10 IU (6 µg), chloramphenicol 30 μg and ampicillin 10 μg standard antibiotic disks were served as positive controls in the assay. In the assay for synergy testing with potassium clavulanate (Macklin, Shanghai, China), positive controls used were penicillin G 10 IU (6 µg) combined with potassium clavulanate and standalone penicillin G 10 IU (6 µg), whereas potassium clavulanate with pure DMSO were used as the negative control for the potassium clavulanate synergism assay. The plates were allowed to stand for about 3 h at 4 °C for pre-diffusion of the extract into the agar and incubated at 37 °C for 24 h. All assays were conducted in triplicate. The diameter of the zone of inhibition around each paper disk was measured to the nearest millimetre (mm), including the size of paper disk [16]. The antibiotic synergistic activities of plant extracts are determined by the growth inhibitory indices (GIIs), which is calculated according to the following formula:

$$\mathrm{G}\mathrm{I}\mathrm{I}\mathrm{s} = \frac{\mathrm{I}\mathrm{Z}\mathrm{D}\,\mathrm{o}\mathrm{f}\,\mathrm{a}\mathrm{n}\mathrm{t}\mathrm{i}\mathrm{b}\mathrm{i}\mathrm{o}\mathrm{t}\mathrm{i}\mathrm{c}\, + \mathrm{p}\mathrm{l}\mathrm{a}\mathrm{n}\mathrm{t}\,\mathrm{e}\mathrm{x}\mathrm{t}\mathrm{r}\mathrm{a}\mathrm{c}\mathrm{t}\,\mathrm{c}\mathrm{o}\mathrm{m}\mathrm{b}\mathrm{i}\mathrm{n}\mathrm{e}\mathrm{d}\,\mathrm{d}\mathrm{i}\mathrm{s}\mathrm{k}}{\mathrm{I}\mathrm{Z}\mathrm{D}\,\mathrm{o}\mathrm{f}\,\mathrm{p}\mathrm{l}\mathrm{a}\mathrm{n}\mathrm{t}\,\mathrm{e}\mathrm{x}\mathrm{t}\mathrm{r}\mathrm{a}\mathrm{c}\mathrm{t}\,\mathrm{a}\mathrm{n}\mathrm{d}\,\mathrm{a}\mathrm{n}\mathrm{t}\mathrm{i}\mathrm{b}\mathrm{i}\mathrm{o}\mathrm{t}\mathrm{i}\mathrm{c}\,\mathrm{i}\mathrm{n} \mathrm{i}\mathrm{n}\mathrm{d}\mathrm{i}\mathrm{v}\mathrm{i}\mathrm{d}\mathrm{u}\mathrm{a}\mathrm{l}\,\mathrm{a}\mathrm{c}\mathrm{t}\mathrm{i}\mathrm{o}\mathrm{n}}$$

The GIIs value > 1 will be considered as synergistic if the inhibition zone of the extract-antibiotic combined disk is greater than their individual agents, when either plant extract and/or the antibiotic used was lacking inhibition zones [17, 18].

### Determination of the minimum inhibitory concentration and minimum bactericidal concentration

The macrobroth dilution assay was carried out on Muller-Hinton broth to determine the minimum inhibitory concentration (MIC) of plant extracts with concentrations ranging from 500–62.5 μg/mL. The MIC for each extract was performed by two-fold serial dilutions (1:2). The reconstituted extract was passed through a 0.22 μm pore size PTFE syringe filter to ensure sterility and the highest extract concentration was set to 1000 μg/mL, which was used to prepare the other extract dilutions. Three individual glass vials filled with clean broth, broth with plant extract and bacteria seeded broth with DMSO were served as controls in the following assay. Each vial starting with the 1000 μg/mL of extract broth mixture was inoculated with 100 μL of the 0.5 McFarland standard bacterial inoculums and incubated at 37 °C for 24 h. The turbidity of each vial was visually observed after incubation and the vial with the least opaqueness will be considered as the MIC.

An aliquot of 100 μL starting from the clear vial that was considered as the MIC was taken and spread on fresh agar plates and incubated to check for microbial growth/colonies. The agar plate free of microbial colonies of the corresponding plant extract concentration was considered as the MBC.

### Determination of the fractional inhibitory concentration and fractional bactericidal concentration

The same macrobroth dilution assay will be carried out on Muller-Hinton broth to determine the fractional inhibitory concentration (FIC), fractional inhibitory concentration index (FICI), fractional bactericidal concentration (FBC) and fractional bactericidal concentration index (FBCI) of plant extract-antibiotic combinations. The FICs and FBCs were determined by MICs and MBCs of the antibiotics that indicated the most significant synergies, respectively. Two-fold serial dilutions (1:2) were performed for both antibiotics and plant extracts tested to obtain a final concentration range of 500–62.5 μg/mL. All standard antibiotic dilutions were separately mixed with the appropriate concentration of chloroform stem, bark and wood extracts in a 1:1 volume ratio, thus obtaining a series of combinations of conventional antibiotics and chloroform plant extracts. The concentrations of combined agents prepared corresponded to 1, 1/2, 1/4, 1/8 of the starting point concentration value of the antibiotics selected for this assay [19]. Four individual glass vials filled with clean broth, broth with plant extract, bacteria seeded broth with DMSO, and bacteria seeded broth with antibiotic were served as controls in the following assay. The FIC/FBC and FICI/FBCI of the antibiotic-plant extract combination agent can be calculated according to the following formula shown below:

$${\mathrm{F}\mathrm{I}\mathrm{C}}_{\mathrm{P}\mathrm{l}\mathrm{a}\mathrm{n}\mathrm{t}\,\mathrm{e}\mathrm{x}\mathrm{t}\mathrm{r}\mathrm{a}\mathrm{c}\mathrm{t}/\mathrm{c}\mathrm{o}\mathrm{m}\mathrm{p}\mathrm{o}\mathrm{u}\mathrm{n}\mathrm{d}\,}= \frac{\mathrm{M}\mathrm{I}\mathrm{C}\,\mathrm{o}\mathrm{f}\,\mathrm{p}\mathrm{l}\mathrm{a}\mathrm{n}\mathrm{t}\,\mathrm{e}\mathrm{x}\mathrm{t}\mathrm{r}\mathrm{a}\mathrm{c}\mathrm{t}\,\mathrm{a}\mathrm{n}\mathrm{d}\,\mathrm{a}\mathrm{n}\mathrm{t}\mathrm{i}\mathrm{b}\mathrm{i}\mathrm{o}\mathrm{t}\mathrm{i}\mathrm{c}\,\mathrm{c}\mathrm{o}\mathrm{m}\mathrm{b}\mathrm{i}\mathrm{n}\mathrm{a}\mathrm{t}\mathrm{i}\mathrm{o}\mathrm{n}}{\mathrm{M}\mathrm{I}\mathrm{C}\,\mathrm{o}\mathrm{f}\,\mathrm{p}\mathrm{l}\mathrm{a}\mathrm{n}\mathrm{t}\,\mathrm{e}\mathrm{x}\mathrm{t}\mathrm{r}\mathrm{a}\mathrm{c}\mathrm{t}/\mathrm{c}\mathrm{o}\mathrm{m}\mathrm{p}\mathrm{o}\mathrm{u}\mathrm{n}\mathrm{d}}$$

$${\mathrm{F}\mathrm{I}\mathrm{C}}_{\mathrm{A}\mathrm{n}\mathrm{t}\mathrm{i}\mathrm{b}\mathrm{i}\mathrm{o}\mathrm{t}\mathrm{i}\mathrm{c} }= \frac{\mathrm{M}\mathrm{I}\mathrm{C}\,\mathrm{o}\mathrm{f}\,\mathrm{p}\mathrm{l}\mathrm{a}\mathrm{n}\mathrm{t}\,\mathrm{e}\mathrm{x}\mathrm{t}\mathrm{r}\mathrm{a}\mathrm{c}\mathrm{t}\,\mathrm{a}\mathrm{n}\mathrm{d}\,\mathrm{a}\mathrm{n}\mathrm{t}\mathrm{i}\mathrm{b}\mathrm{i}\mathrm{o}\mathrm{t}\mathrm{i}\mathrm{c}\,\mathrm{c}\mathrm{o}\mathrm{m}\mathrm{b}\mathrm{i}\mathrm{n}\mathrm{a}\mathrm{t}\mathrm{i}\mathrm{o}\mathrm{n}}{\mathrm{M}\mathrm{I}\mathrm{C}\,\mathrm{o}\mathrm{f}\,\mathrm{a}\mathrm{n}\mathrm{t}\mathrm{i}\mathrm{b}\mathrm{i}\mathrm{o}\mathrm{t}\mathrm{i}\mathrm{c}}$$

$$\begin{aligned}&{\mathrm{F}\mathrm{I}\mathrm{C} \mathrm{i}\mathrm{n}\mathrm{d}\mathrm{e}\mathrm{x}}_{\mathrm{P}\mathrm{l}\mathrm{a}\mathrm{n}\mathrm{t}\,\mathrm{e}\mathrm{x}\mathrm{t}\mathrm{r}\mathrm{a}\mathrm{c}\mathrm{t}/\mathrm{c}\mathrm{o}\mathrm{m}\mathrm{p}\mathrm{o}\mathrm{u}\mathrm{n}\mathrm{d}\,\mathrm{a}\mathrm{n}\mathrm{t}\mathrm{i}\mathrm{b}\mathrm{i}\mathrm{o}\mathrm{t}\mathrm{i}\mathrm{c}\,\mathrm{c}\mathrm{o}\mathrm{m}\mathrm{b}\mathrm{i}\mathrm{n}\mathrm{a}\mathrm{t}\mathrm{i}\mathrm{o}\mathrm{n}}\\& = {\mathrm{F}\mathrm{I}\mathrm{C}}_{\mathrm{P}\mathrm{l}\mathrm{a}\mathrm{n}\mathrm{t}\,\mathrm{e}\mathrm{x}\mathrm{t}\mathrm{r}\mathrm{a}\mathrm{c}\mathrm{t}/\mathrm{c}\mathrm{o}\mathrm{m}\mathrm{p}\mathrm{o}\mathrm{u}\mathrm{n}\mathrm{d} }+ {\mathrm{F}\mathrm{I}\mathrm{C}}_{\mathrm{A}\mathrm{n}\mathrm{t}\mathrm{i}\mathrm{b}\mathrm{i}\mathrm{o}\mathrm{t}\mathrm{i}\mathrm{c}} \end{aligned}$$

FIC index ≤ 0.5 will be considered as synergistic, > 0.5 but < 1 as partially synergistic, additive when = 1, in different when > 1 but < 2 and ≥ 2 as antagonistic [20, 21].

### Screening for qualitative phytochemical analysis

About 3 mg/3 mL of chloroform, methanol and *n*-hexane extracts of *C. bracteosus* were qualitatively screened for the presence of bioactive secondary metabolites like flavonoids, phenols, saponins, and tannins.

### Test for flavonoids

Approximately 3 mL of the filtrated extract was treated with a few consecutive drops of 10% 2 N NaOH (Sigma-Aldrich, St. Louis, USA) solution. The formation of yellow colour was an indication of flavonoids present in the extract [22].

### Test for phenols

Approximately 3 mL of the filtrated extract was treated with a few consecutive drops of 5% ferric chloride (Sigma-Aldrich, St. Louis, USA) solution. The formation of dark blue to black colour was an indication of phenols present in the extract [22].

### Froth test for saponins

Approximately 5 mL of the filtrated extract was subjected to vigorous shaking for 15 min. A foamy generation of at least 1 cm in height was an indication of saponins present in the extract [22].

### Braemer’s test for tannins

Approximately 3 mL of the filtrated extract was treated with consecutive drops of 5% ferric chloride solution. The formation of dark blue or greenish black colour was an indication of tannins present in the extract [22].

### Test for coumarins

Approximately 3 mL of the filtrated extract was treated with a few consecutive drops of 10% 2 N NaOH solution. The formation of yellow colour was an indication of coumarins present in the extract [22].

### Test for alkaloids

Approximately 3 mL of the filtrated extract was treated with 1 mL of Dragendorff's reagent (Sigma-Aldrich, St. Louis, USA). The presence of alkaloids will be indicated by a reddish-brown or brick-red colour appearance [22].

### Thin layer chromatography of crude extracts

The chloroform extracts of *C. bracteosus* were subjected to Thin Layer Chromatography** (**TLC) for separation of phytochemical compounds in fractions.

About 100 mg of the dried crude plant extract was reconstituted with 1 mL of ethanol (Merck, New Jersey, United States) and filtered through cotton and anhydrous magnesium sulphate. Two (2) μL aliquots of the filtered plant extract reconstituted with absolute ethanol were consecutively transferred to Silica Gel 60 F_254_ coated aluminium-backed TLC plates (5 × 10 cm) (Merck, New Jersey, United States) at one minute intervals using a 10 μL microcapillary tube. The crude reconstituted extracts were spotted on the TLC plates and allowed to dry. Seventy (70) % *n*-hexane + 30% ethyl acetate was used as the eluent in the TLC assay. The fully dried TLC plate was subjected to visible light, UV wavelength 254 nm, UV wavelength 365 nm, vanillin + sulphuric acid spray (test for terpenes), 10% ferric chloride spray (tannin test) and 10% sodium hydroxide (flavonoids test) and Dragendoff's reagent (Sigma-Aldrich, St. Louis, USA) spray (alkaloid test) to visualize compound separations in fractions that were indicated by spots on the TLC plate. The presence of tannins in the bands of separated fraction will be indicated by the colour appearance of dark blue-greenish black [23]. The presence of alkaloids will be indicated by a reddish-brown or brick-red colour appearance [24]. The Rf value of each band was calculated by dividing the distance travelled by the spot demarcated for the compound by the distance travelled by band demarcated for the solvent from the point of origin at the mobile phase. A good solvent system has the ability to move compounds between the Rf value of 0.2 and 0.4 [25].

### Fractionation of crude extracts

Larger quantities of fractions from chloroform crude extracts of stem, bark and wood were separated using preparative Silica Gel 60 F254 coated glass-backed TLC plates (20 × 20 cm) (Merck, New Jersey, United States) under the same eluent that composed of 70% *n*-hexane + 30% ethyl acetate. For the preparative TLC assay, 10 μL aliquots of crude plant extract reconstituted with absolute ethanol were consecutively and timely transferred on to plates and allowed to be fully dried. The experiment was repeated five times. The silica composed of separated fractions, was then scrapped from TLC plates and collected into individual centrifuge tubes (50 mL). Absolute ethanol was used to macerate the compound impregnated silica and sonicated for several hours using ultra-sonicator (Labline, Mumbai, India) at 40ºC. The sonicated mixture was then subjected to ultracentrifugation using a refrigerated centrifuge (Eppendorf, Hamburg, Germany) at 9000 rpm for 10 min for utter separation of compounds from silica in the mixture. The silica precipitate free supernatant was obtained and filtered with 0.22 μm pore size PTFE syringe filter. The ethanol content present in the filtered supernatant was evaporated using a rotary evaporator under vacuum at 40 °C and dried completely. Similarly, the Rf value of each band was recorded after running preparative TLC. The dried fractions were numbered from baseline (bottom) to solvent font (top) of each TLC plate according to their appearance and later evaluated for antibacterial, antibiotic synergistic activity and subjected for GC–MS and HPLC analysis.

### Assay for the screening of antibacterial activity and antibiotic synergistic activity of extract fractions

The in vitro agar paper disk diffusion assay was used to evaluate the antibacterial and antibiotic synergistic activities of fractions obtained from chloroform plant extracts of bark, stem and wood. Organisms and antibiotics that responded most effectively for crude chloroform stem, wood and bark extracts of *C. bracteosus* were taken for analysing antibacterial and antibiotic synergistic activities of these fractions. The assay was performed on Muller-Hinton agar (Oxoid, Hampshire, UK). A sterile cotton swab was immersed in a bacterial suspension of 0.5 McFarland turbidity standards for each test organism and swabbed over the entire agar surface for even distribution. The dried filtrates composed of fractions were reconstituted with absolute DMSO. Aliquots equivalent to 30 μL of the reconstituted filtrates were transferred individually to sterilised filter paper disks (Whatman Grade 3; GE Healthcare, Chicago, USA) of 6 mm in diameter. Furthermore, penicillin G 10 IU (6 µg) and ampicillin 10 μg disks (Oxoid, Hampshire, UK) were loaded and impregnated with 30 μL of the reconstituted filtrates for synergistic activity testing [15]. The impregnated disks were allowed to dry for few hours.

The paper disks were placed on the agar plate using a sterilised forceps. The paper disks impregnated with pure DMSO was considered as the negative control and penicillin G 10 IU (6 µg), ampicillin 10 μg and gentamicin 10 μg standard antibiotic disks were served as positive controls in the assay. The plates were allowed to stand for about 3 h at 4 °C for pre-diffusion of the extract into the agar and incubated at 37 °C for 24 h. The assay was conducted in triplicates. The diameter of the zone of inhibition around each paper disk was measured to the nearest millimetre (mm), including the size of the paper disk. The antibiotic synergistic activities of fractions are determined by GIIs.

### Gas chromatography-mass spectrometry (GC–MS)

The fractions of stem, bark, wood extracts were individually dissolved in absolute ethanol (Merck, New Jersey, United States) and GC–MS analysis was performed on a PerkinElmer Clarus 680, 5Q85 mass spectrometer (Massachusetts, USA) using the Elite 5MS column (30 m × 0.25 mm × 0.25 µm film). An electron ionisation system with an ionisation energy of 70 eV was used for GC–MS detection. Scan range 35–600 m/z in mode of 0.5 scans s^−1^. The carrier gas applied was helium with a flow rate of 1 mL/min. Injector and oven temperatures were set to 250 °C. The oven temperature was the same as with the GC analysis. The samples of 1 µL were injected in the 50:1 split mode.

The identification of components was performed by comparison of their RRT (relative retention time), peak area %, percentage abundance and mass spectra-to-charge ratio with those of authentic samples, literature/data and computerised MS–data bank NIST (National Institute of Standards and Technology) 2.0 (2005) database. The peak area method was followed for the quantitative determination of different constituents, and the percentage was calculated relatively.

### High-Performance Liquid Chromatography (HPLC)

The TLC fraction number 6 of *C. bracteosus* chloroform bark extract, which exhibited both standalone antibacterial activity and synergistic activity with ampicillin and penicillin G was dissolved in dichloromethane (DCM) (Merck, New Jersey, United States) for optimal compound detection based on polarity and passed through a 0.22 μm pore size PTFE syringe filter for reversed-phase HPLC analysis. Prior to subjecting the sample for HPLC separation, the column was washed with absolute DCM. Subsequently, the chromatographic fractionation was performed using the Zorbax Eclipse technology of C-18 column (Massachusetts, USA) accommodated by the Agilent 1200 series HPLC system (Santa Clara, USA). Sixty (60) μL of the sample was injected at a flow rate of 0.6 mL/min to achieve a retention time 20 min per run. Absolute acetonitrile was used as the standard and as the solvent of choice during the mobile phase in the analysis. The RT peaks in the HPLC chromatograms were compared with compound references of digoxigenin, where the UV wavelength was set at 218–220 nm [26] and lauric acid by setting the UV wavelength at 230–256 nm [27] for abundance indication of the aforesaid compounds in the isolated fraction.

### Field emission scanning electron microscopic (FE-SEM) characterisation

Both plant extract treated and non-treated bacterial cells of *B. cereus*, *B. subtilis* and *A. baumannii* were examined under FE-SEM for the comparison and assessment of their external structures. Bacteria were grown on agar plates, and 12 mm round sterilised coverslips were placed on their single colonies and pressed gently for cell attachment. This procedure was carried out for both non-treated reference samples and plant extract treated samples. Subsequently, *C. bracteosus* stem, bark and wood extract treated bacteria were directly taken from the inhibition zones for each organism. Both treated and non-treated samples were fixed overnight with 3% (v/v) glutaraldehyde (Sigma-Aldrich, St. Louis, USA) at 4 °C. The fixed samples were removed of glutaraldehyde and washed thrice for 10 min each time in 0.1 M phosphate buffer solution (Sigma-Aldrich, St. Louis, USA) and dehydrated with ethanol series 30%, 50%, 70%, 80%, 90%, 96% for 10 min each and with absolute ethanol twice for 10 min each. After dehydration in alcohol, the samples were critically dried in ethanol and hexamethyldisilazane (HMDS) (Sigma-Aldrich, St. Louis, USA) (2:1) for 15 min, ethanol and HMDS (1:2) for 15 min and finally, HMDS alone for 15 min. The 100% HMDS step was repeated and the excess HMDS was removed, leaving just a layer covering the coverslip to fully dry under the fume hood overnight. The fully dried samples on coverslips were mounted on metal stubs and sputter coated with gold for 1 min using Quorum Q150R Plus (Judges Scientific plc, London, UK) and microscopically analysed by FEI Quanta 400 F model EDX: INCA 400 Oxford instrument with X-Max Detector FE-SEM (Brno, Czech Republic) for exterior morphological alterations.

### Statistical data analysis

The data obtained for antibacterial and synergistic antibiotic assays were statistically evaluated using One-way ANOVA, and post-hoc tests Dunnett's and Tukey HSD were applied for multiple comparisons between samples and controls and among samples, respectively. The data analysis was performed using GraphPad Prism software version 9.4.1 (San Diego, California, USA) to compare the IZD (inhibition zone diameter) means of each group with the IZD means of each other group. The analysed data were considered to be statistically significant when the p-value of less than 0.05 (P < 0.05) at a confidence level of 95%.

## Results and discussion

A variety of medicinal plants used in folklore medicine are rich sources of natural antibacterial products. In such, their properties contributing to antimicrobial action have been used to treat various infections in humans caused by pathogenic bacteria for many years. Plentiful evidence exists about the efficiency and universality of herbs used in folklore medicine through their constant harnessing and application. Hence, the present pioneering study revealed the antibacterial and antibiotic potentiating ability of *C. bracteosus* extracts, particularly against the CDC classified superbug *A. baumannii* [28].

### Yield of plant extracts

The highest yields obtained for *C. bracteosus* extracts include leaves methanol (0.762%), chloroform leaves (0.761%) and leaves *n*-hexane (0.75%). The raw masses and the final yields *C. bracteosus* extracts are summarised in Table S1.

### Assessment for antibacterial activity and antibiotic synergistic activity

**Fig. 2:**
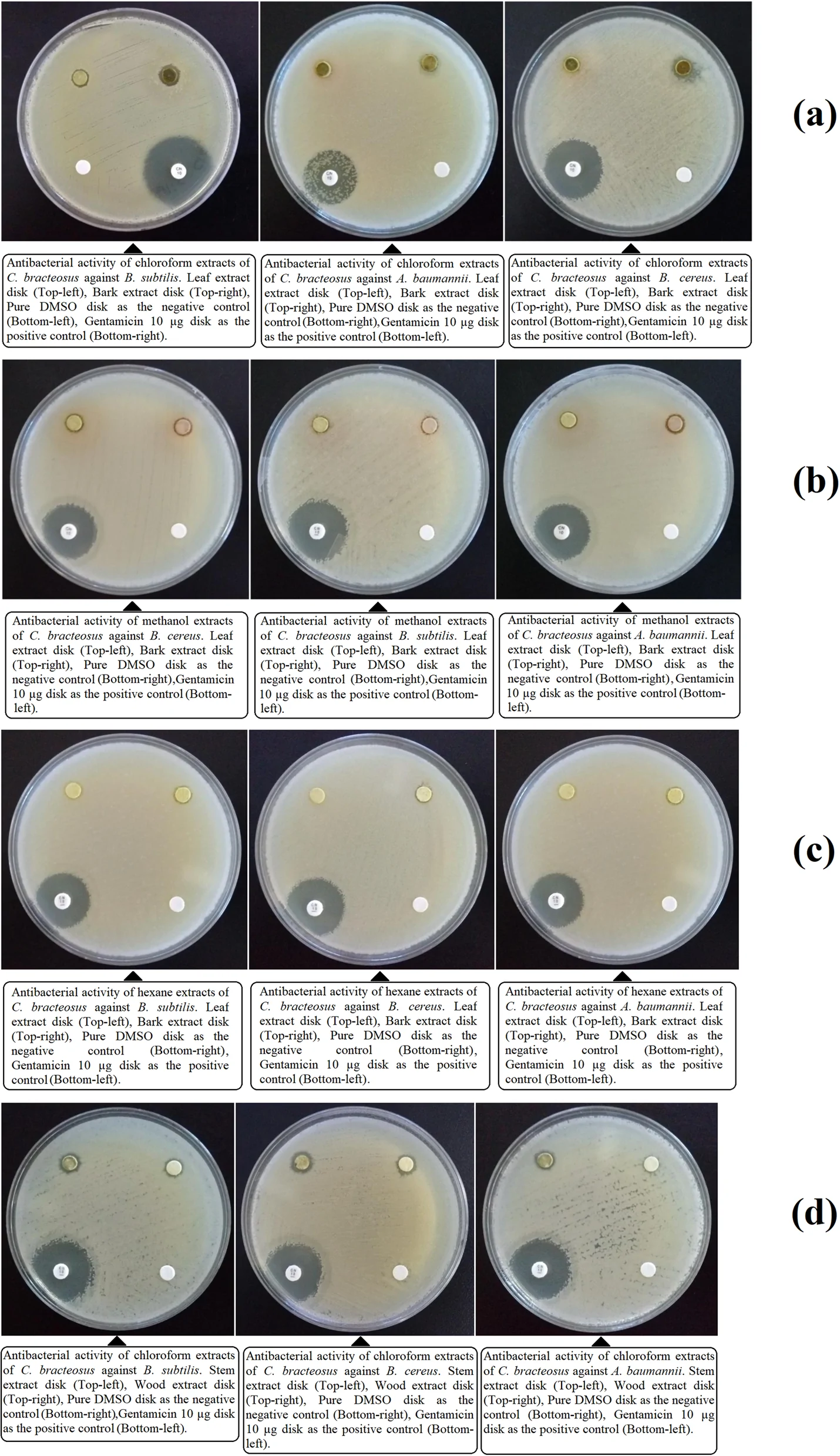
Standalone antibacterial activity of *C. bracteosus* crude extracts of chloroform (**a**) and (**d**), methanol (**b**) and hexane (**c**)

**Fig. 3:**
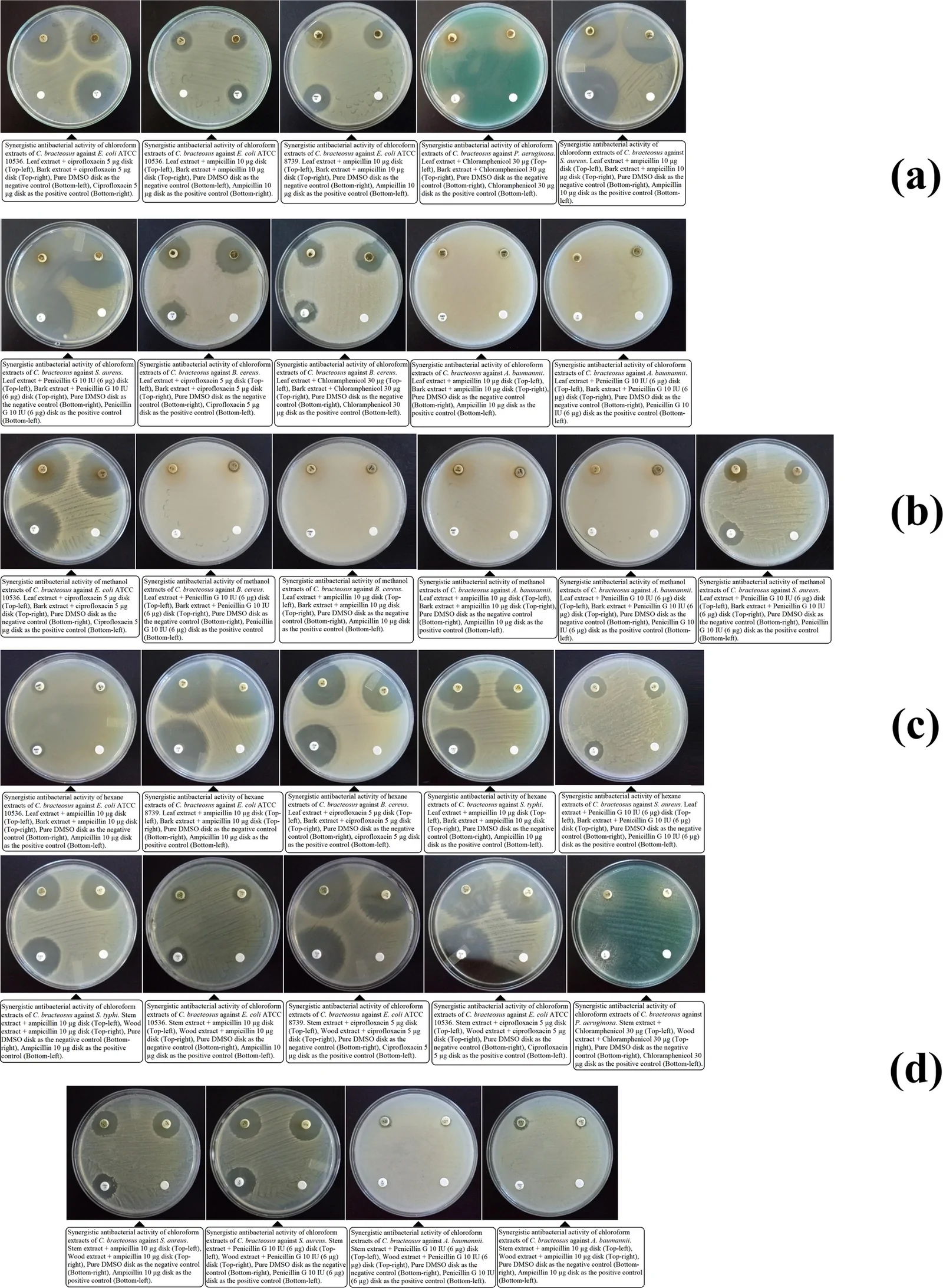
Antibiotic synergistic activity of *C. bracteosus* crude extracts of chloroform (**a**) and (**d**), methanol (**b**) and hexane (**c**)

In the present study, the results of the antibacterial activity assay indicating the zone of inhibition for standalone disks containing leaves, bark, stem and wood of *C. bracteosus* extracted in chloroform, methanol and *n*-hexane against *B. subtilis, B. cereus, S. aureus, P. aeruginosa, S. typhi, E. coli* and *A. baumannii* are summarised in Table 1, Tables S2-S3 and Fig. 2. Whereas, results recorded for inhibition zones around disks containing *C. bracteosus* leaves, bark, stem and wood extracts combined with standard antibiotics in the assay for synergistic antibiotic activity against the above tested bacterial isolates are presented in Tables 2 and 3, Tables S4-S5 and Fig. 3. The DMSO negative control disk did not indicate any observable antibacterial activity in all assays, while inhibition zones of significant diameters were detected around standard antibiotic disks with the exception of penicillin G against *E. coli, P. aeruginosa, B. subtilis, B. cereus* and *A. baumannii* and ampicillin against *B. subtilis, B. cereus* and *A. baumannii* that severed as positive controls. The data obtained in the assays will be elucidated below.

**Table 1:** Diameters for the zone of inhibition for chloroform extracts of *C. bracteosus* against bacteria in the disc diffusion antibacterial assay

Bacterial isolates			Zone of inhibition (mm)			
	Leaves extract	Bark extract	Stem extract	Wood extract	Gentamicin 10 µg (PC)	Pure DMSO (NC)
*E. coli* ATCC 10536	0	0	0	0	19.5 ± 0.32	0
*B. subtilis* ATCC 6633	7.3 ± 0.72	8.6 ± 0.27	9.5 ± 0.28	9 ± 0.28	26.6 ± 0.24	0
*S. aureus* ATCC 11632	0	0	0	0	24 ± 0.56	0
*B. cereus* ATCC 10876	0	9 ± 0.28	10 ± 0.26	9.5 ± 0.29	28 ± 0.56	0
*P. aeruginosa* ATCC 10145	0	0	0	0	18.5 ± 0.78	0
*S. typhi* ATCC 6539	0	0	0	0	19 ± 0.80	0
*E. coli* ATCC 8739	0	0	0	0	21 ± 0.57	0
*A. baumannii* ATCC 19606	6.2 ± 0.03	6.5 ± 0.58	9 ± 0.57	7.5 ± 0.54	21 ± 0.54	0

*PC* positive control, *NC* negative control, Extract concentration is at 1000 µg/disc, Values are represented as the mean of triplicates (*n* = 3) ± S.E.M

**Table 2:** Diameters for the zone of inhibition for chloroform leaves and bark extracts of *C. bracteosus* in the disc diffusion synergistic antibacterial assay

Bacterial isolates		Zone of inhibition (mm)				GIIs	
		Standard antibiotic disc with potency	Standard antibiotic disc	Standard antibiotic and extract combination disc			
				Leaves	Bark	Leaves	Bark
*E. coli* ATCC 10536		Levofloxacin 5 µg	32 ± 0.56	31 ± 0. 57	28 ± 0.88	-	-
		*Ciprofloxacin 5 µg	31 ± 0.53	31 ± 0. 67	*32 ± 0.56	1	1.03
		Penicillin G 10 IU (6 µg)	0	0	0	-	-
		Gentamicin 10 µg	19.5 ± 0.87	17 ± 0.78	15 ± 0.46	-	-
		Chloramphenicol 30 µg	28 ± 0.47	27.5 ± 0.69	27 ± 0.87	-	-
		*Ampicillin 10 µg	14.5 ± 0.57	*15 ± 0.66	*16 ± 0.64	1.03	1.10
*E. coli* ATCC 8739		Levofloxacin 5 µg	36 ± 0.58	36 ± 0.75	36 ± 0.77	-	-
		Ciprofloxacin 5 µg	40 ± 0.54	37 ± 0.58	40 ± 0.71	-	-
		Penicillin G 10 IU (6 µg)	0	0	0	-	-
		Gentamicin 10 µg	25 ± 0.57	18 ± 0.58	22 ± 0.60	-	-
		Chloramphenicol 30 µg	34 ± 0.52	33 ± 0.67	33.5 ± 0.89	-	-
		*Ampicillin 10 µg	19 ± 0.56	19 ± 0.58	*20 ± 0.57	1	1.05
*S. typhi* ATCC 6539		Levofloxacin 5 µg	34.5 ± 0.55	24 ± 0.57	30 ± 0.59	-	-
		Ciprofloxacin 5 µg	39 ± 0.23	31.5 ± 0.44	34 ± 0.53	-	-
		Penicillin G 10 IU (6 µg)	17 ± 0.58	15 ± 0.51	15 ± 0.57	-	-
		Gentamicin 10 µg	18 ± 0.66	16 ± 0.59	18 ± 0.54	-	-
		Chloramphenicol 30 µg	31 ± 0.57	26 ± 0.58	29 ± 0.33	-	-
		Ampicillin 10 µg	26 ± 0.88	23 ± 0.23	21 ± 0.13	-	-
*P. aeruginosa* ATCC 10145		Levofloxacin 5 µg	23 ± 0.66	19 ± 0.57	22.5 ± 0.59	-	-
		Ciprofloxacin 5 µg	31.5 ± 0.57	28 ± 0.88	30 ± 0.34	-	-
		Penicillin G 10 IU (6 µg)	0	0	0	-	-
		Gentamicin 10 µg	19.5 ± 0.58	7.5 ± 0.89	10 ± 0.45	-	-
		*Chloramphenicol 30 µg	0	0	*8 ± 0.57	-	> 1
		Ampicillin 10 µg	0	0	0	-	-
*B. subtilis* ATCC 6633		Levofloxacin 5 µg	26 ± 0.58	26 ± 0.43	26 ± 0.45	-	-
		Ciprofloxacin 5 µg	28 ± 0.57	24 ± 0.62	27 ± 0.68	-	-
		Penicillin G 10 IU (6 µg)	0	0	0	-	-
		Gentamicin 10 µg	21 ± 0.84	16 ± 0.34	19 ± 0.23	-	-
		Chloramphenicol 30 µg	19.5 ± 0.35	19 ± 0.51	22 ± 0.57	-	-
		Ampicillin 10 µg	0	0	0	-	-
*S. aureus* ATCC 11632	Levofloxacin 5 µg		31 ± 0.46	29 ± 0.53	30.5 ± 0.77	-	-
	Ciprofloxacin 5 µg		29 ± 0.57	25 ± 0.33	29 ± 0.16	-	-
	*Penicillin G 10 IU (6 µg)		44 ± 0.67	*47 ± 0.51	*48 ± 0.57	1.07	1.09
	Gentamicin 10 µg		24 ± 0.76	20 ± 0.43	18.5 ± 0.24	-	-
	Chloramphenicol 30 µg		31 ± 0.35	29 ± 0.63	28 ± 0.18	-	-
	*Ampicillin 10 µg		41 ± 0.53	*42 ± 0.58	*43 ± 0.52	1.02	1.05
*B. cereus* ATCC 10876	Levofloxacin 5 µg		25 ± 0.54	26 ± 0.51	27 ± 0.57	-	-
	*Ciprofloxacin 5 µg		18 ± 0.56	*23 ± 0.44	*28 ± 0.23	1.28	1.04
	Penicillin G 10 IU (6 µg)		0	0	0	-	-
	Gentamicin 10 µg		19 ± 0.69	14.5 ± 0.54	19 ± 0.57	-	-
	*Chloramphenicol 30 µg		16 ± 0.55	*19 ± 0.52	20 ± 0.57	1.19	-
	Ampicillin 10 µg		0	0	0	-	-
*A. baumannii* ATCC 19606	Levofloxacin 5 µg		26 ± 0.58	25 ± 0.52	26 ± 0.78	-	-
	Ciprofloxacin 5 µg		28.5 ± 0.47	27 ± 0.57	29 ± 0.94	-	-
	*Penicillin G 10 IU (6 µg)		0	0	*8.5 ± 0.51	-	1.3
	Gentamicin 10 µg		21.5 ± 0.51	17 ± 0.57	20 ± 0.58	-	-
	Chloramphenicol 30 µg		23 ± 0.55	20 ± 0.50	22 ± 0.42	-	-
	*Ampicillin 10 µg		0	*6.5 ± 0.58	*8 ± 0.92	1.05	1.23

Extract concentration is at 1000 µg/disc, Values for the zone of inhibition are represented as the mean of triplicates (*n* = 3) ± S.E.M, *GIIs* Growth inhibitory indices (synergy index). *Indicates data for antibiotic synergism

**Table 3:** Diameters for the zone of inhibition for chloroform stem and wood extracts of *C. bracteosus* in the disc diffusion synergistic antibacterial assay

Bacterial isolates	Zone of inhibition (mm)				GIIs	
	Standard antibiotic disc with potency	Standard antibiotic disc	Standard antibiotic and extract combination disc			
			Stem	Wood	Stem	Wood
*E. coli* ATCC 10536	Levofloxacin 5 µg	32 ± 0.56	31 ± 0. 57	28 ± 0.88	-	-
	*Ciprofloxacin 5 µg	38 ± 0.54	38 ± 0. 65	*40 ± 0.58	-	1.05
	Penicillin G 10 IU (6 µg)	0	0	0	-	-
	Gentamicin 10 µg	19.5 ± 0.87	17 ± 0.78	15 ± 0.46	-	-
	Chloramphenicol 30 µg	28 ± 0.47	27 ± 0.69	27 ± 0.87	-	-
	*Ampicillin 10 µg	12 ± 0.57	12 ± 0. 66	*13 ± 0.63	-	1.08
*E. coli* ATCC 8739	Levofloxacin 5 µg	36 ± 0.58	36 ± 0.75	36 ± 0.77	-	-
	*Ciprofloxacin 5 µg	38.5 ± 0.54	*39.5 ± 0.58	*39 ± 0.71	1.02	1.01
	Penicillin G 10 IU (6 µg)	0	0	0	-	-
	Gentamicin 10 µg	25 ± 0.57	19 ± 0.58	22 ± 0.60	-	-
	Chloramphenicol 30 µg	34 ± 0.52	33 ± 0.67	34 ± 0.89	-	-
	Ampicillin 10 µg	17 ± 0.57	17 ± 0.58	17 ± 0.57	-	**-**
*S. typhi* ATCC 6539	Levofloxacin 5 µg	34.5 ± 0.55	25 ± 0.57	30 ± 0.59	-	-
	Ciprofloxacin 5 µg	39 ± 0.23	32.5 ± 0.44	34 ± 0.53	-	-
	Penicillin G 10 IU (6 µg)	17 ± 0.58	15 ± 0.51	15.5 ± 0.57	-	-
	Gentamicin 10 µg	18 ± 0.66	17 ± 0.59	18 ± 0.54	-	-
	Chloramphenicol 30 µg	31 ± 0.57	26 ± 0.58	29 ± 0.33	-	-
	*Ampicillin 10 µg	26 ± 0.88	*27 ± 0.23	*27 ± 0.13	1.04	1.04
*P. aeruginosa* ATCC 10145	Levofloxacin 5 µg	23 ± 0.66	19 ± 0.57	22.5 ± 0.59	-	-
	Ciprofloxacin 5 µg	31.5 ± 0.57	29 ± 0.88	31 ± 0.34	-	-
	Penicillin G 10 IU (6 µg)	0	0	0	-	-
	Gentamicin 10 µg	19.5 ± 0.58	7 ± 0.89	10 ± 0.45	-	-
	*Chloramphenicol 30 µg	0	0	*9 ± 0.55	-	> 1
	Ampicillin 10 µg	0	0	0	-	-
*B. subtilis* ATCC 6633	Levofloxacin 5 µg	29 ± 0.58	30 ± 0.43	30.5 ± 0.45	-	-
	Ciprofloxacin 5 µg	29 ± 0.57	31 ± 0.62	28.5 ± 0.68	-	-
	Penicillin G 10 IU (6 µg)	0	0	0	-	-
	Gentamicin 10 µg	21 ± 0.84	16 ± 0.34	19 ± 0.23	-	-
	Chloramphenicol 30 µg	19.5 ± 0.35	19 ± 0.51	22 ± 0.57	-	-
	Ampicillin 10 µg	0	0	0	-	-
*S. aureus* ATCC 11632	Levofloxacin 5 µg	31 ± 0.46	29 ± 0.53	30.5 ± 0.77	-	-
	Ciprofloxacin 5 µg	29 ± 0.57	25 ± 0.33	29 ± 0.16	-	-
	*Penicillin G 10 IU (6 µg)	21.5 ± 0.67	*23 ± 0.51	*23 ± 0.57	1.07	1.07
	Gentamicin 10 µg	24 ± 0.76	20 ± 0.43	18.5 ± 0.24	-	-
	Chloramphenicol 30 µg	31 ± 0.35	29 ± 0.63	28 ± 0.18	-	-
	*Ampicillin 10 µg	18 ± 0.53	*20 ± 0.58	*20 ± 0.52	1.1	1.1
*B. cereus* ATCC 10876	Levofloxacin 5 µg	30 ± 0.54	31 ± 0.51	31 ± 0.57	-	-
	Ciprofloxacin 5 µg	33 ± 0.56	33 ± 0.44	32.5 ± 0.23	-	-
	Penicillin G 10 IU (6 µg)	0	10 ± 0.19	8 ± 0.19	-	-
	Gentamicin 10 µg	19 ± 0.69	14.5 ± 0.54	19 ± 0.57	-	-
	Chloramphenicol 30 µg	17 ± 0.55	19 ± 0.52	20 ± 0.57	-	-
	Ampicillin 10 µg	0	0	0	-	-
*A. baumannii* ATCC 19606	Levofloxacin 5 µg	26 ± 0.58	25 ± 0.52	26 ± 0.78	-	-
	Ciprofloxacin 5 µg	28.5 ± 0.47	27 ± 0.57	29 ± 0.94	-	-
	*Penicillin G 10 IU (6 µg)	0	*10 ± 0.51	*8 ± 0.51	1.11	1.07
	Gentamicin 10 µg	21.5 ± 0.51	17 ± 0.57	20 ± 0.58	-	-
	Chloramphenicol 30 µg	23 ± 0.55	20 ± 0.50	22 ± 0.42	-	-
	*Ampicillin 10 µg	0	*11 ± 0.58	*10 ± 0.92	1.22	1.33

Extract concentration is at 1000 µg/disc, Values for the zone of inhibition are represented as the mean of triplicates (n = 3) ± S.E.M, *GIIs* Growth inhibitory indices (synergy index). *Indicates data for antibiotic synergism

Chloroform leaves, bark, stem and wood extracts of *C. bracteosus* indicated observable yet mild antibacterial activity against *B. cereus, B. subtilis* and *A. baumannii,* with stem extract having superior antibacterial activity. The chloroform leaf extract induced mean IZDs of 7.3 ± 0.72 mm for *B. subtilis* and 6.2 ± 0.03 mm for *A. baumannii,* and the chloroform bark extract induced mean IZDs of 8.6 ± 0.27 mm for *B. subtilis*, 9 ± 0.28 mm for *B. cereus* and 6.5 ± 0.58 mm for *A. baumannii* at their highest potency of 1000 µg in disk (Fig. 2a). The chloroform stem extract induced mean IZDs of 9.5 ± 0.28 mm for *B. subtilis*, 10 ± 0.26 mm for *B. cereus* and 9 ± 0.57 mm for *A. baumannii* and the chloroform wood extract induced mean IZDs of 9 ± 0.28 mm for *B. subtilis*, 9.5 ± 0.29 mm for *B. cereus* and 7.5 ± 0.54 mm for *A. baumannii* at their highest potency of 1000 µg in disk (Fig. 2d). The standalone chloroform extracts of the plant did not indicate any observable antibacterial activity for the other bacterial strains tested. However, antibiotic synergistic activity of chloroform extracts was observed for multiple strains tested, with the exception of *S. typhi* for leaf and bark extracts, *B. subtilis* and *B. cereus* for stem and wood extracts. Also, the chloroform stem extract did not show antibiotic synergism against *B. cereus.*

In the synergistic antibacterial activity assay against *E. coli* ATCC 10536, the chloroform leaf extract with ampicillin disk induced a zone of inhibition approximately 0.5 mm larger than the standard ampicillin disk in diameter (Fig. 3a). Whereas, the chloroform bark extract exhibited inhibition zones that were approximately 1.5 mm larger than the standard ampicillin disk (Fig. 3a) and approximately 1 mm larger than the standard ciprofloxacin disk in diameter, respectively (Fig. 3a). The chloroform wood extract exhibited inhibition zones that were approximately 1 mm larger than the standard ampicillin disk (Fig. 3d) and approximately 2 mm larger than the standard ciprofloxacin disk in diameters respectively (Fig. 3d). The chloroform bark extract with ampicillin combined disk had the highest synergistic activity with a GIIs synergy index of 1.10 for *E. coli* ATCC 10536. Antibiotic synergism was observed for *E. coli* ATCC 8739 when the chloroform bark extract with ampicillin disk exhibited an inhibition zone that was approximately 1 mm larger than the standard ampicillin disk (Fig. 3a), and chloroform stem and wood extracts with inhibition zones approximately 1 mm and 0.5 mm larger than the standard ciprofloxacin disk in diameters, respectively (Fig. 3d). The highest GIIs synergy index of 1.05 was detected for ampicillin-bark combined disk against *E. coli* ATCC 8739.

Both chloroform bark and wood extracts potentiated the antibacterial action of chloramphenicol, which induced an inhibition zone of 8 ± 0.57 mm and 9 ± 0.55 mm, respectively in diameter for *P. aeruginosa* when combined, whereas the standard chloramphenicol disk did not show any zone of inhibition for the bacterium (Fig. 3d). A GIIs synergy index > 1 was obtained for chloramphenicol-bark and chloramphenicol-wood extract combined disk against *P. aeruginosa*.

In the synergistic antibacterial activity assay against *S. aureus*, the chloroform leaf extract with ampicillin disk induced a zone of inhibition approximately 1 mm larger than the standard ampicillin disk in diameter (Fig. 3a). Whereas, the chloroform bark extract exhibited inhibition zones that were approximately 3 mm larger than the standard ampicillin disk (Fig. 3a) and chloroform stem and wood extracts with inhibition zones approximately 2 mm larger than the ampicillin disk in diameters, respectively (Fig. 3d). Penicillin G also showed synergy against *S. aureus* when used in combination with chloroform leaf and bark extracts, in which the antibiotic and the leaf extract combined disk induced an inhibition zone approximately 3 mm larger than the standard penicillin G disk. Meanwhile, the penicillin G and bark extract combined disk induced an inhibition zone approximately 4 mm larger than the standard penicillin G disk (Fig. 3a). Both chloroform stem and wood extracts also showed synergy with penicillin G against *S. aureus* with inhibition zones 1.5 mm larger than the standard penicillin G disk (Fig. 3d). The chloroform bark extract with penicillin G combined disk had the highest synergistic activity with a GIIs synergy index of 1.09 for *S. aureus*.

Significant antibiotic synergism was observed for *B. cereus* when the chloroform leaf and bark extracts with ciprofloxacin combined disks exhibited inhibition zones that were approximately 5 mm and 10 mm larger than the standard ciprofloxacin disk, respectively. (Fig. 3a). The chloroform extracts of *C. bracteosus* also showed significant synergies for chloramphenicol against *B. cereus*, in which the leaf extract-chloramphenicol combined disk induced an inhibition zone approximately 3 mm larger than the chloramphenicol standard disk and the bark extract-chloramphenicol combined disk induced an inhibition zone approximately 4 mm larger than the chloramphenicol standard disk (Fig. 3a). The chloroform bark extract with ciprofloxacin combined disk had the chloroform bark extract with ciprofloxacin combined disc had the largest IZD in synergistic activity with a GIIs synergy index of 1.04 for *B. cereus*.

Remarkably, the chloroform extracts of *C. bracteosus* noticeably potentiated the antibacterial action of ampicillin and penicillin G against *A. baumannii*. The chloroform leaf-ampicillin combined disk induced an inhibition zone of 6.5 ± 0.58 mm, and the chloroform bark-ampicillin combined disk induced an inhibition zone of 8 ± 0.92 mm in diameter for *A. baumannii*, whereas the standard ampicillin disk did not show any zone of inhibition for the bacterium (Fig. 3a). Chloroform stem and wood extracts also showed observable synergies when combined with ampicillin, which indicated inhibition zones of 11 ± 0.58 mm and 10 ± 0.92 mm in diameter, respectively (Fig. 3d). An inhibition zone of 8.5 ± 0.51 mm in diameter was detected for the chloroform bark extract-penicillin G combined disk against *A. baumannii*, whereas the standard penicillin G disk did not show any zone of inhibition for the bacterium (Fig. 3a). Furthermore, chloroform stem and wood extracts significantly synergised penicillin G when combined, which induced inhibition zones with diameters of 10 ± 0.51 mm and 8 ± 0.51 mm, respectively (Fig. 3d). A GIIs synergy index of 1.33 was indicated for *A. baumannii* when the chloroform wood extract and ampicillin were used in combination, which was overall the highest GIIs in the experiment.

**Fig. 4:**
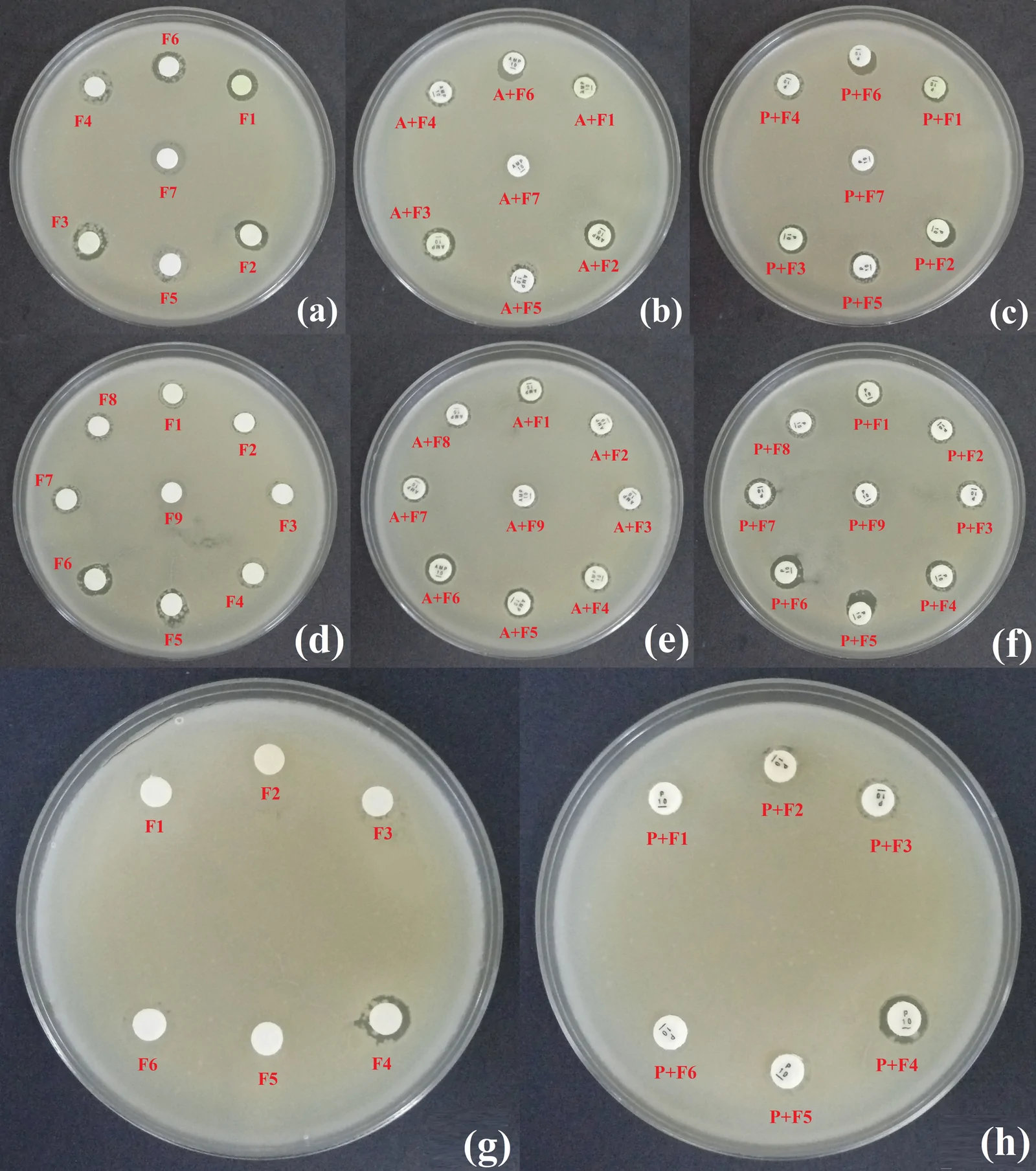
Antibacterial and antibiotic synergistic activities of separated fractions of *C. bracteosus* chloroform extracts against *A. baumannii*. Standalone antibacterial action of bark fractions (**a**), Ampicillin synergistic action of bark fractions (**b**), Penicillin G synergistic action of bark fractions (**c**), Standalone antibacterial action of stem fractions (**d**), Ampicillin synergistic action of stem fractions (**e**), Penicillin G synergistic action of stem fractions (**f**), Standalone antibacterial action of wood fractions (**g**), Penicillin G synergistic action of wood fractions (**h**), “F” represents fraction, “A + F’ represents Ampicillin 10 µg disk + isolated fraction, “P + F’ represents Penicillin G 10 IU (6 µg disk) + isolated fraction

**Fig. 5:**
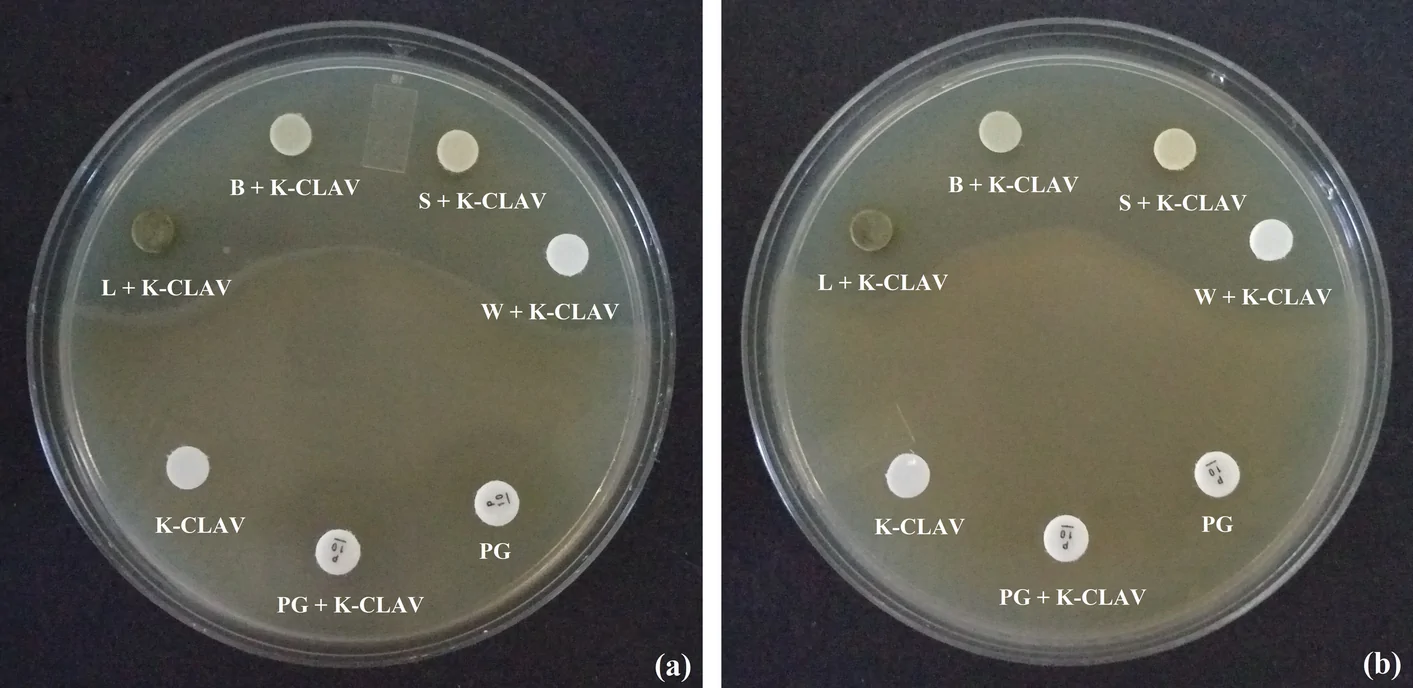
Synergistic activities of chloroform leaves, bark, stem and wood extracts of *C. bracteosus* with potassium clavulanate against *S. typhi* (**a**) and against *E. coli* (ATCC 10536) (**b**). L + K-CLAV = leaves with potassium clavulanate), B + K-CLAV = bark with potassium clavulanate), S + K-CLAV = stem with potassium clavulanate), W + K-CLAV = wood with potassium clavulanate), K-CLAV = potassium clavulanate in DMSO (negative control), PG + K-CLAV = Penicillin G 10 IU (6 µg disk) with potassium clavulanate (positive control), PG = Penicillin G 10 IU (6 µg disk) (positive control)

Chloroform bark, stem and wood extracts indicated the MIC at 500 µg/mL for *B. cereus*, *A. baumannii* and *B. subtilis*. However, there was no bactericidal activity observed for any of the extracts, even at their highest concentration of 1000 µg/mL, as bacterial colonies were absent in the MBC assay. The chloroform leaf extract did not indicate an MIC since bacterial growth was observed at all concentrations tested. Chloroform stem, bark and wood extracts indicated significant synergy with ampicillin and penicillin G for *A. baumannii* in the FIC assay with a FICI of < 0.5. The MICs for ciprofloxacin and chloramphenicol were 15 µg/mL and 2.5 µg/mL for *B. cereus,* respectively, and an additive effect was observed for chloroform bark extract with ciprofloxacin and chloramphenicol against *B. cereus* in the FIC assay*,* which indicated a FICI of 1. The MICs for chloroform plant extracts, standard antibiotics, and FICs, FICIs, FBCs and FBCIs for plant extract-antibiotic combinations are summarised in Table 4. Both ciprofloxacin and chloroform bark extract with ciprofloxacin combination indicated bactericidal activity in the FBC assay. Similar results were obtained for MIC, FIC, FICI, MBC, FBC and FBCI for chloroform stem and wood extracts. Furthermore, the results for standalone antibacterial activity and antibiotic synergistic activities of fractions of bark, stem and wood extracts of *C. bracteosus* against *A. baumannii* are summarised in Table 5 and Fig. 4. Four fractions isolated from chloroform crude bark extract (fractions 1–3 and 6) indicated antibacterial activity against *A. baumannii* and two fractions showed antibiotic synergism against the same bacterium. Fraction 1 of chloroform bark showed the highest standalone antibacterial activity against *A. baumannii* with an inhibition zone of 9.5 ± 0.43 mm (Fig. 4a). Fractions 4 and 6 of chloroform stem extract evenly synergised penicillin G and ampicillin against *A. baumannii* (Fig. 4e and f)*.* Four fractions* (*Fractions 5–8) of *C. bracteosus* chloroform stem extract showed inhibition zones against *A. baumannii* with fraction 6 of chloroform stem indicating the highest inhibition zone diameter of 9 ± 0.45 mm (Fig. 4d). Fractions 1,2 and 4 and fractions 1–9 of *C. bracteosus* chloroform stem extract showed synergism with ampicillin and penicillin G, respectively (Fig. 4e and f). Fraction 1 of chloroform stem indicated the highest antibiotic synergism for ampicillin with an inhibition zone of 8 ± 0.43 mm and GIIs synergy indices of > 1. Meanwhile, Fraction 6 of chloroform stem indicated the highest antibiotic synergism for penicillin G with an inhibition zone of 10 ± 0.38 mm and GIIs synergy indices of 1.05 (Fig. 4f). Only one fraction (fraction 4) of *C. bracteosus* chloroform wood extract showed an inhibition zone diameter of 9 ± 0.58 mm against *A. baumannii.* The same fraction synergised penicillin G with an inhibition zone of 9 ± 0.58 mm and GIIs synergy indices of 1.06 (Fig. 4g and h). Meanwhile, fractions 1–4 and 9 of chloroform stem, fractions 4, 6 and 7 of chloroform bark, and fractions 1–3 and 5–7 of chloroform wood did not show any observable standalone antibacterial activity when tested against *A. baumannii*. Potassium clavulanate significantly synergized the antibacterial activities of chloroform leaf, bark, stem and wood extracts of *C. bracteosus* with IZDs of 22 ± 0.34 mm, 28 ± 0.54 mm, 27.5 ± 0.65 mm and 21 ± 0.58 mm respectively for *S. typhi* as shown in Fig. 5(a) and 19 ± 0.58 mm, 26 ± 0.38 mm, 27 ± 0.56 mm and 23 ± 0.43 mm respectively for *E. coli* (ATCC 10536) as shown in Fig. 5b. However, no observable antibacterial activity was detected against other organisms tested, including both positive and negative controls tested in the assay. Also, no synergistic activity was detected for *B. cereus, B. subtilis* and *A. baumannii* when the aforesaid extracts were combined with potassium clavulanate, unlike their antibiotic combination. Our study also indicates that *C. bracteosus* chloroform extracts can be potentiated by potassium clavulanate against certain Gram-negative bacteria, in which potassium clavulanate was a β-beta-lactamase inhibitor [29].

**Table 4:** Data obtained for fractional inhibitory concentration and fractional bactericidal concentration for chloroform extracts of *C. bracteosus*

Antibacterial agent	MIC (µg/mL)		MBC (µg/mL)		FIC (µg/mL)		FICI		FBC (µg/mL)		FBCI	
	Ab	Bc	Ab	Bc	Ab	Bc	Ab	Bc	Ab	Bc	Ab	Bc
CB	500	500	-	-	NA	NA	NA	NA	NA	NA	NA	NA
CS	500	500	-	-	NA	NA	NA	NA	NA	NA	NA	NA
CW	500	500	-	-	NA	NA	NA	NA	NA	NA	NA	NA
CIP	-	2.5	-	5	NA	1	NA	NA	NA	NA	NA	NA
CH	-	30	-	-	NA	1	NA	NA	NA	NA	NA	NA
AMP	-	-	-	-	NA	NA	NA	NA	NA	NA	NA	NA
PEG	-	-	-	-	NA	NA	NA	NA	NA	NA	NA	NA
CB + CIP	-	2.5	-	5	-	0.005	-	1	-	1	-	2
CB + CH	-	15	-	-	-	0.03	-	1	-	-	-	-
CB + AMP	250	-	-	-	< 0.5	-	< 0.5	-	-	-	-	-
CB + PEG	250	-	-	-	< 0.5	-	< 0.5	-	-	-	-	-
CS + AMP	250	-	-	-	< 0.5	-	< 0.5	-	-	-	-	-
CS + PEG	250	-	-	-	< 0.5	-	< 0.5	-	-	-	-	-
CW + AMP	250	-	-	-	< 0.5	-	< 0.5	-	-	-	-	-
CW + PEG	250	-	-	-	< 0.5	-	< 0.5	-	-	-	-	-

*MIC* Minimum Inhibitory Concentration, *MBC* Minimum Bactericidal Concentration, *FIC* Fractional Inhibitory Concentration, *FICI* Fractional Inhibitory Concentration Index, *FBC* Fractional Bactericidal Concentration, *FBC* Fractional Bactericidal Concentration Index, *Ab A. baumannii* ATCC 19606, *Bc B. cereus* ATCC 10876, *CB* Chloroform bark extract, *CS* Chloroform stem extract, *CW* Chloroform wood extract, *CIP* Ciprofloxacin 5 µg, *CH* Chloramphenicol 30 µg, *AMP* Ampicillin 10 µg, *PEG* Penicillin G 10 IU (6 µg), *CB* + *CIP* Chloroform bark extract + Ciprofloxacin 5 µg, *CB* + *CH* Chloroform bark extract + Chloramphenicol 30 µg, *CB* + *AMP* Chloroform bark extract + Ampicillin 10 µg, Chloroform bark extract + Penicillin G 10 IU (6 µg), *NA* Not applicable

**Table 5:** Antibacterial and antibiotic synergistic activities of fractions from chloroform extracts against *A. baumannii*

Type	Agent tested	Zone of Inhibition (mm)	GIIs
Stem	F1	0	-
	F2	0	-
	F3	0	-
	F4	0	-
	F5	9 ± 0.65	-
	F6	9.5 ± 0.60	-
	F7	8 ± 0.45	-
	F8	8 ± 0.56	-
	F9	0	-
	*A + F1	8 ± 0.43	> 1
	*A + F2	7 ± 0.22	> 1
	A + F3	0	-
	*A + F4	7 ± 0.66	> 1
	A + F5	8 ± 0.67	-
	A + F6	9 ± 0.62	-
	A + F7	8 ± 0.46	-
	A + F8	8 ± 0.45	-
	A + F9	0	-
	*P + F1	8 ± 0.38	> 1
	*P + F2	8 ± 0.24	> 1
	*P + F3	8.5 ± 0.54	> 1
	*P + F4	9 ± 0.76	> 1
	*P + F5	9.5 ± 0.62	1.06
	*P + F6	10 ± 0.38	1.05
	*P + F7	9 ± 0.57	1.13
	*P + F8	8.5 ± 0.76	1.06
	*P + F9	8 ± 0.56	> 1
Bark	F1	9.5 ± 0.43	-
	F2	9 ± 0.64	-
	F3	9 ± 0.36	-
	F4	0	-
	F5	0	-
	F6	7 ± 0.33	-
	F7	0	-
	A + F1	7 ± 0. 37	-
	A + F2	9 ± 0. 75	-
	A + F3	8 ± 0. 36	-
	*A + F4	7.3 ± 0. 45	> 1
	A + F5	0	-
	*A + F6	7 ± 0. 34	> 1
	A + F7	0	-
	P + F1	8 ± 0. 65	-
	P + F2	8.5 ± 0. 54	-
	P + F3	8.5 ± 0. 34	-
	*P + F4	8 ± 0. 52	> 1
	P + F5	0	0
	*P + F6	8 ± 0.56	> 1
	P + F7	0	-
Wood	F1	0	-
	F2	0	-
	F3	0	-
	F4	9 ± 0.58	**-**
	F5	0	-
	F6	0	-
	F7	0	-
	A + F1	0	-
	A + F2	0	-
Wood	A + F3	0	-
	A + F4	0	-
	A + F5	0	-
	A + F6	0	-
	A + F7	0	-
	P + F1	0	-
	P + F2	0	-
	P + F3	0	-
	*P + F4	9.5 ± 0.58	1.06
	P + F5	0	-
	P + F6	0	-
	P + F7	0	-

Values for the zone of inhibition are represented as the mean of triplicates (*n* = 3) ± S.E.M, F = Isolated fraction of extract, *A* Ampicillin, *P* Penicillin G, *GIIs* Growth inhibitory indices (synergy index). *Indicates data for antibiotic synergism

The methanol leaf extract of *C. bracteosus* indicated observable yet mild antibacterial activity against *B. cereus, B. subtilis* and *A. baumannii*, whereas the methanol bark extract did not indicate any observable antibacterial activity. The methanol leaf extract induced mean IZDs of 7 ± 0.71 mm for *B. subtilis* and 7 ± 0.70 mm for *B. cereus* and 7 ± 0.77 mm for *A. baumannii* at its highest potency of 1000 µg in disk (Fig. 2b). The standalone methanol extracts of the plant did not indicate any observable antibacterial activity for the other bacterial strains tested. However, antibiotic synergistic activity of methanol extracts was observed for *E. coli*, *S. aureus* and *A. baumannii*. The best antibiotic synergism was observed for *A. baumannii*.

Antibiotic synergism was observed for *E. coli* ATCC 8739 when the methanol leaf extract with ciprofloxacin disk exhibited an inhibition zone that was approximately 1 mm larger than the standard ciprofloxacin disk with a GIIs synergy index of 1.03 (Fig. 3b). In the synergistic antibacterial activity assay against *S. aureus*, the methanol leaf extract with penicillin G disk induced a zone of inhibition approximately 1.5 mm larger than the standard ampicillin disk in diameter (Fig. 3b). Whereas the methanol bark extract exhibited inhibition zones that were approximately 1 mm larger than the standard penicillin G disk (Fig. 3b). The methanol leaf extract with penicillin G combined disk had the highest synergistic activity out of the two with a GIIs synergy index of 1.09 for *S. aureus*.

The methanol extracts of *C. bracteosus* markedly potentiated the antibacterial action of ampicillin and penicillin G against *A. baumannii*. The methanol leaf-ampicillin combined disk, and the chloroform bark-ampicillin combined disk induced an inhibition zone of 7.5 ± 0.57 mm in diameter for *A. baumannii*, whereas the standard ampicillin disk did not show any zone of inhibition for the bacterium (Fig. 3b). An inhibition zone of 8± 0.52 mm in diameter was detected for the methanol bark extract-penicillin G combined disk against *A. baumannii*, whereas the standard penicillin G disk did not show any zone of inhibition for the bacterium (Fig. 3b). A GIIs synergy index > 1 was obtained for the chloroform bark extract of *C. bracteosus* when combined with penicillin G against *A. baumannii*. A remarkable antibiotic potentiating effect is also observed when methanolic bark extracts of *C. bracteosus* were combined with ampicillin and penicillin G against *B. cereus*, which indicated GIIs synergy indices > 1. The methanol bark-ampicillin combined disk induced an inhibition zone of 6.3 ± 0.44 mm in diameter for *B. cereus*, whereas the standard ampicillin disk did not show any zone of inhibition for the bacterium (Fig. 3b). The methanol bark extract with penicillin G combined disk induced an inhibition zone of 8.5 ± 0.53 mm for *B. cereus,* whereas the standard penicillin G disk did not show any zone of inhibition for the bacterium (Fig. 3b). Antibiotic synergism was not detected for *E. coli* ATCC 10536, *S. typhi*, *P. aeruginosa* and *B. subtilis* for methanolic extracts of *C. bracteosus.*

Both *n*-hexane leaf and bark extracts of *C. bracteosus* indicated observable yet mild antibacterial activity against *B. cereus, B. subtilis* and *A. baumannii* with bark extract having superior antibacterial activity. The *n*-hexane leaf extract induced mean IZDs of 6.1 ± 0.71 mm and the *n*-hexane bark extract induced mean IZDs of 7 ± 0.29 mm for *B. cereus* and 8 ± 0.45 mm for *A. baumannii* at their highest potency of 1000 µg in disk (Fig. 2c). The standalone *n*-hexane extracts of the plant did not indicate any observable antibacterial activity for the other bacterial strains tested. However, antibiotic synergistic activity of *n*-hexane extracts was observed for multiple strains tested, with the exception of *B. subtilis*, *P. aeruginosa* and *A. baumannii*. The best antibiotic synergism was observed for *E. coli* and *S. aureus*.

In the synergistic antibacterial activity assay against *E. coli* ATCC 10536, the *n*-hexane leaf extract with ampicillin disk induced a zone of inhibition approximately 2 mm larger than the standard ampicillin disk in diameter (Fig. 3c). Whereas, the *n*-hexane bark extract exhibited an inhibition zone that was approximately 1 mm larger than the standard ampicillin disk (Fig. 3c). The highest GIIs synergy index was obtained for *n*-hexane leaf extract of *C. bracteosus* when combined with ampicillin against *E. coli* ATCC 10536, which was 1.2. Both *n*-hexane leaf and bark extract-ciprofloxacin combined disks exhibited inhibition zones that were approximately 3.5 mm larger than the standard ciprofloxacin disk in diameter for *E. coli* ATCC 8739 with a GIIs synergy index of 1.09 (Fig. 3c). Similarly, both *n*-hexane leaf and bark extract-ampicillin combined disks exhibited inhibition zones that were approximately 3.5 mm larger than the standard ciprofloxacin disk in diameter for *S. typhi* with a GIIs synergy index of 1.07 (Fig. 3c).

In the synergistic antibacterial activity assay against *S. aureus*, penicillin G showed synergy against *S. aureus* when used in combination with *n*-hexane leaf and bark extracts, in which penicillin G-leaf extract and penicillin G-bark extract combined disks induced an inhibition zone approximately 2.5 mm larger than the standard penicillin G disk with a GIIs synergy index of 1.18 (Fig. 3c). Antibiotic synergism was observed for *B. cereus* when the *n*-hexane leaf extract of *C. bracteosus* with ciprofloxacin combined disk exhibited inhibition zones that were approximately 2 mm larger than the standard ciprofloxacin disk (Fig. 3c). The *n*-hexane leaf extract with ciprofloxacin combined disk indicated a GIIs synergy index of 1.07 for *B. cereus*. Antibiotic synergism is not detected for *S. typhi*, *P. aeruginosa*, *B. subtilis*, *A. baumannii* for *n-*hexane extracts of *C. bracteosus.*

Similarly, other species of *Cleistanthus* have shown antimicrobial activities against a multitude of pathogens. A study revealed that petroleum ether, chloroform and acetone leaf and stem extracts of *C. collinus* indicated antibacterial activity against *E. coli* NCIM 2065. Meanwhile, the chloroform stem extract induced a zone of inhibition of 10.2 ± 0.1 in diameter [10]. Another study indicates that *n*-hexane extracts of *C. collinus* were active against *E. coli* (MTCC-433), which indicated inhibition zones with diameters of 39 mm and 7 mm for serial and direct dilutions respectively [30]. There are no direct studies involving the investigation of phytochemical constituents of *C. bracteosus* and their mechanisms of action on the inhibition of bacterial activity as of yet. The results of this study revealed that Gram-positive bacteria like *B. subtilis* are more susceptible to the phytochemical molecules present in the plant extract than Gram-negative bacteria like *E. coli*. Conceivably, this was due to the structure of the Gram-positive cell wall, which makes the bacterium more permeable to phytochemical molecules. Meanwhile, the lipopolysaccharide layer present in the outer membranes of Gram-negative bacteria encases the bacterium by acting as a permeability barrier, thus restricting the diffusion of larger bioactive compounds derived from plants. Hence, this mediates to a higher degree of resistance to antibiotic agents than Gram-positive bacteria [31, 32]. *E. coli* has a number of genes responsible for mediating resistance to antibacterial agents through the expression of efflux pumps, activation of antibacterial molecule degrading enzymes and the modification of antibacterial molecule binding sites, leading to the inhibition of DNA gyrase and DNA topoisomerase IV synthesis [33]. Meanwhile, bacteria like *S. aureus* can express genes that are responsible for generating alternate pathways associated with metabolic activity in order to circumvent metabolic blocks imposed by antibacterial compounds targeted against bacterial metabolites. This type of resistance can be seen in MRSA as their cell walls are programmed to produce PBP mutants like PBP2 to PBP5, which can restrict the interaction of the antibacterial molecule with the bacterium [34]. *A. baumannii* too showed resistance to certain extracts of *C. bracteosus* and standard antibiotics like ampicillin and penicillin used in the experiment. Intriguingly, this nature of drug resistance in *A. baumannii* can arise due to the presence of heteroresistant bacterial cells with efflux pumps expressed by genes like *adeA* and *adeS* [35, 36].

The antibacterial activity of the plant extracts in our study was an indication of the presence of broad or narrow-spectrum antibiotic compounds. Meanwhile, the qualitative phytochemical analysis in this study revealed the presence of bioactive secondary metabolites like flavonoids, phenols, coumarins, saponins and tannins in the extracts of *C. bracteosus*, which may have individually or synergistically mediated the antibacterial activity and the enhancement of antibiotic potentiation against the selected organisms. Arguably, the lack of MIC for chloroform leaf extract could be due to the tested bacteria being highly resistant to its sole inhibitory effects. Hence, the following study suggests chloroform plant extracts are capable of exhibiting antibacterial activity, and thus the combination of natural compounds from plants with synthetically derived antibiotics was a fine strategy to combat antimicrobial resistance. The presence of synergistic activity between the plant extract and the standard antibiotic that led to the inhibition of bacterial activity was an indication that phytochemical compounds are capable of facilitating the penetration of conventional antibiotics into bacteria by modifying their action mechanism pathways to reverse antimicrobial resistance. Relatively, one such potential molecule related to our study was hexadecenoic acid induced antibacterial activity against MDR strains of *S. aureus* W35, *P. aeruginosa* D31, *K. pneumoniae* DF30, and *K. pneumonia* [37]. hexadecenoic acid was a monosaturated fatty acid that was capable of synergizing vancomycin against *Enterococcus faecalis* (VRE), which lowered the MIC from 32–64 μg/mL [38]. Statistical analysis in the study indicated that chloroform extracts against *B. subtilis, B. cereus, A. baumannii* attained a *p*-value < 0.05 (*p* = 0.000015) when compared with the control (gentamicin 10 µg) (Sup. Figure 1). Hence, the means of IZDs for the aforesaid data obtained were statistically significant.

### Phytochemical profile of *C. bracteosu*s extracts

**Fig. 6:**
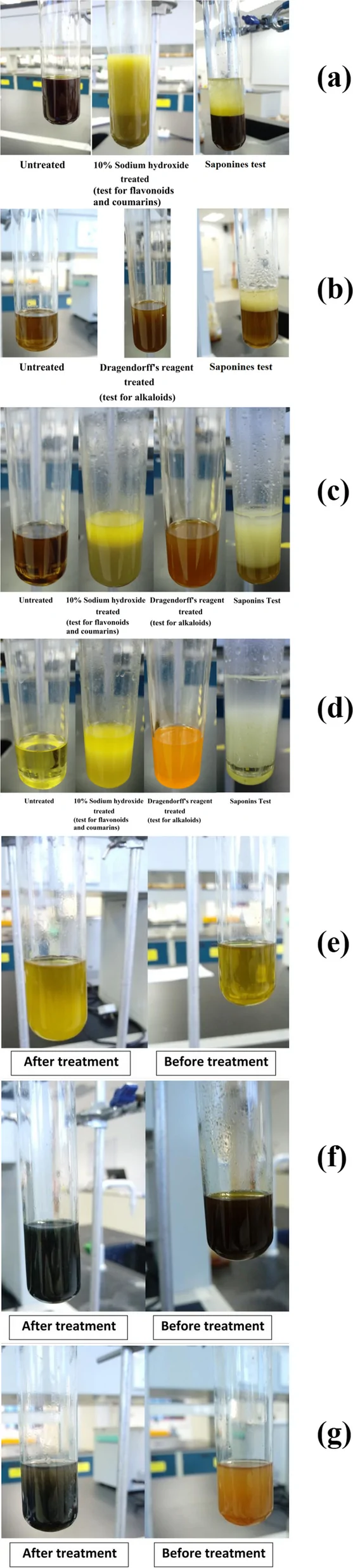
Qualitative phytochemical analysis of *C. bracteosus* crude extracts of chloroform: leaves (**a**), bark (**b**), stem (**c**) and wood (**d**), hexane bark tested for flavonoids/coumarins (**e**), and methanol: leaves (**f**) and bark (**g**) tested for phenols/tannins

Chloroform, methanol and *n*-hexane extracts of *C. bracteosus* leaves, bark, stem and wood indicated the presence of bioactive secondary metabolites like flavonoids, phenols, alkaloids, tannins, coumarins and saponins in the qualitative phytochemical analysis test. The results for phytochemical analysis of *C. bracteosus* extracts are summarised in Table S6 and Fig. 6. Our chemical analysis of the extracts revealed which resonates from the study done by Mohanta et al. and Elangomathavan et al. [39, 40]. Phytochemical compounds like tannins, terpenoids, flavonoids, and alkaloids exhibit inhibitory action against the activity of bacteria [32]. Moreover, these phytochemical compounds are capable of suspending the growth, replication and function of bacteria by the inhibition of cell wall and protein synthesis, perturbation of their plasma membrane, modulation/interference of pathways associated with metabolism and gene expression that are essential for nucleic acid synthesis [41]. Meanwhile, the phytochemical compound known as thiophene has the ability to mediate antibacterial activity against *B. subtilis* by disrupting the polymerisation of the bacterial protein called filamenting temperature-sensitive mutant Z (FtsZ), which was responsible for the growth of the bacterium [42].

### Thin layer chromatography compound analysis

**Fig. 7:**
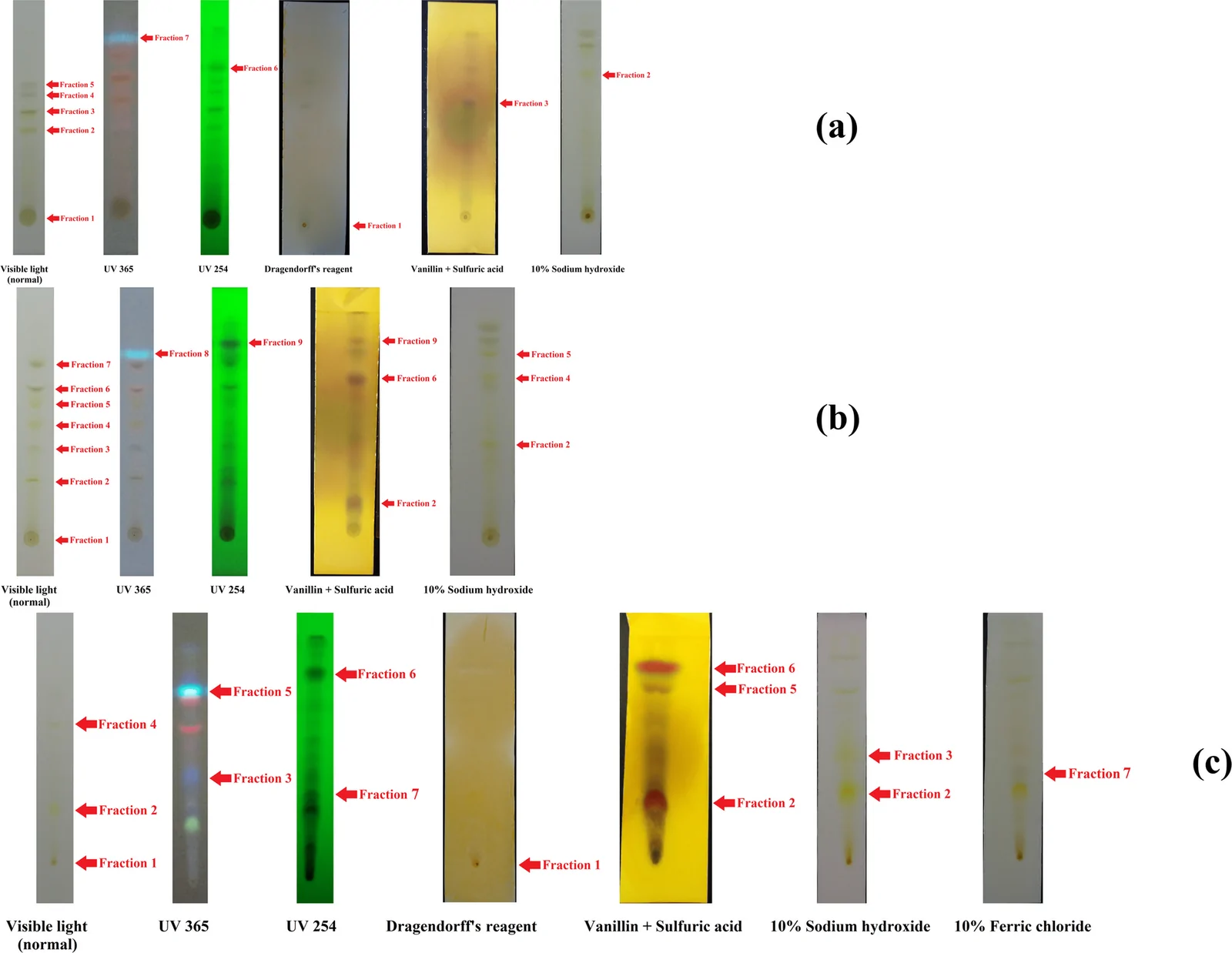
TLC fractions of chloroform (**a**) bark, (**b**) stem and (**c**) wood extracts of *C. bracteosus*

Results recorded for TLC of *C. bracteosus* chloroform extracts are summarized in Table S7-S9 and illustrated in Fig. 7. Both crude bark and stem chloroform extracts of *C. bracteosus* produced several bands under different settings, indicating multiple fractions of isolated compounds. Most bands were visible under UV 365 for the chloroform bark extract and UV 254 for the chloroform stem extract. Band number 2 of bark and band number 3 of stem chloroform extracts appeared after spraying ferric chloride, which indicated the presence of tannins in their respective separated fractions. Meanwhile, the band number 1 of the stem chloroform extract turned reddish-brown in colour when sprayed with Dragendorff's reagent, which was an indication of alkaloids present in the separated fraction. Furthermore, the band number 3 of bark, band numbers 2, 6 and 9 of stem, and band numbers 2, 5 and 6 of wood indicated the presence of terpenes in the fractions when sprayed with vanillin + sulphuric acid. In the preparative TLC assay, approximately 9 fractions from chloroform stem, 7 fractions from chloroform bark and the wood extracts of *C. bracteosus* were separated.

### Chemical composition of *C. bracteosus*

**Fig. 8:**
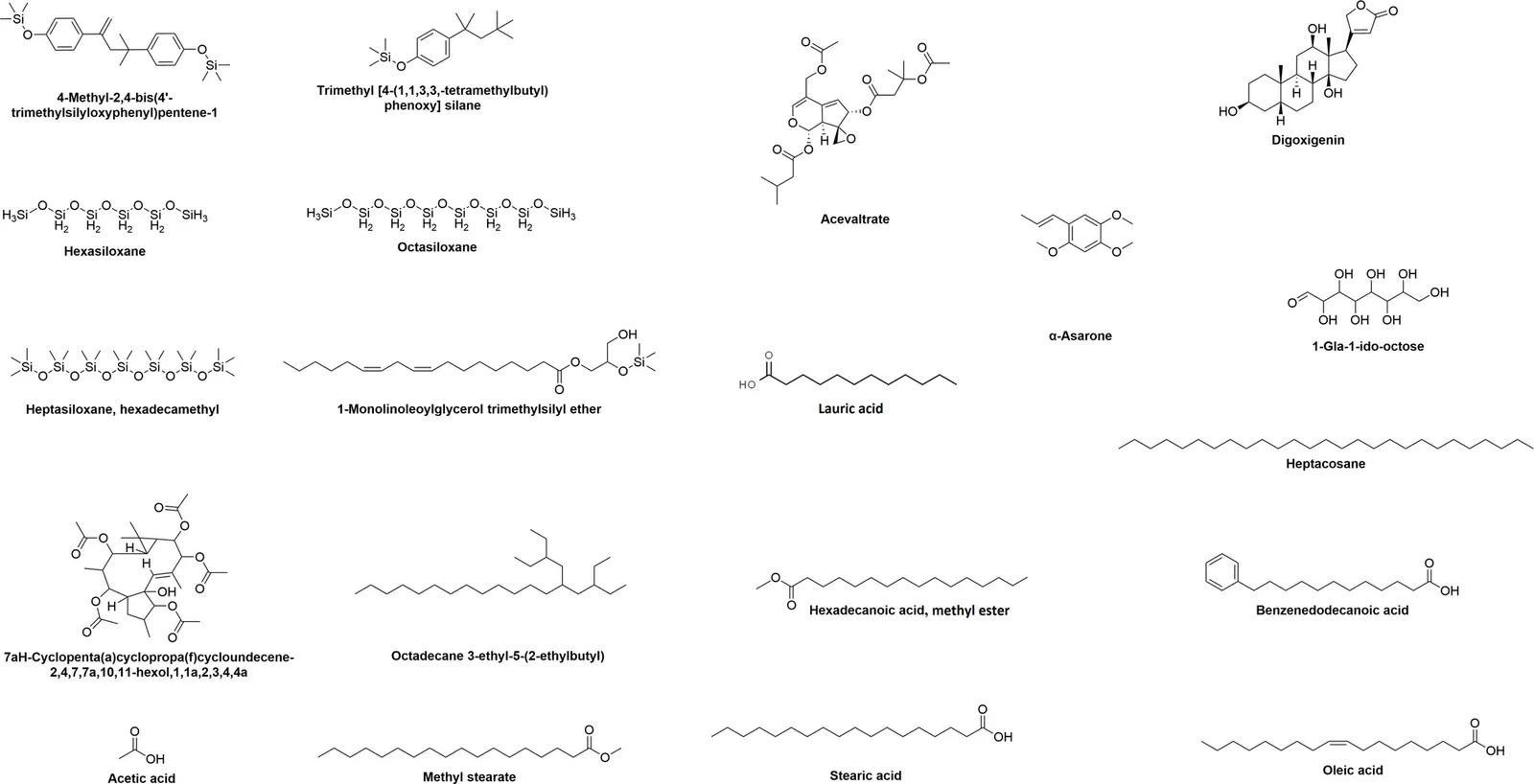
Chemical structures of bioactive compounds present in *C. bracteosus* chloroform stem, bark and wood fractions

The fractions of *C. bracteosus* chloroform extracts represent 23 phytochemical compounds according to the data obtained in GC–MS analysis (Table S10 and Sup. Figure 2). Major compounds that are bioactive in these extracts include methyl stearate, trimethyl [4-(1,1,3,3,-tetramethylbutyl) phenoxy] silane, palmitic acid or hexadecanoic acid, hexasiloxane, oleic acid, 1-Monolinoleoylglycerol trimethylsilyl ether, 9,12,15-Octadecatrienoic acid, 7aH-Cyclopenta(a)cyclopropa(f)cycloundecene-2,4,7,7a,10,11-hexol,1,1a,2,3,4,4a, 4-Methyl-2,4-bis(4'-trimethylsilyloxyphenyl) pentene-1, 1-Gala-1-ido-octose, lauric acid or dodecanoic acid, octadecane 3-ethyl-5-(2-ethylbutyl)-, acevaltrate, digoxigenin, stearic acid and α-Asarone (Fig. 8). Further analysis with HPLC implied the presence of digoxigenin (RT 4.512 min) detected at UV wavelength of 218–220 nm and lauric acid (RT 6.772 min) detected at UV wavelength of 230–256 nm in TLC isolated fraction number 6 of chloroform bark extract, which principally exist as credible compounds contributing to antibacterial and antibiotic synergism in *C. bracteosus* (Sup. Figure 3) [43, 44]. Meanwhile, an investigation showed that *Ophiorrhiza rugosa* var. prostrata (D.Don) & Mondal composed of methyl stearate, a methyl ester fatty acid that showed antibacterial activity against *B. subtilis, S. typhi* and *E. coli* [45]. Lydia et al. demonstrated the antibacterial potential of octadecanamide, a carboximidic acid found in *M. charantia* fruit extract against *Bacillus licheniformis* [46]. *A. karoo* leaf extract containing Trimethyl [4-(1,1,3,3,-tetramethylbutyl) phenoxy] silane showed antibacterial activity against *S*. *aureus* and *Proteus vulgaris* [47, 48]. Mbakazi et al. showed the antibacterial action of hexasiloxane present in *Sarcophyte sanguine* against *S. aureus* and *E. coli* [49]*.* A study conducted by Naeim et al. indicated that octasiloxane present in *Cordyline pumilio* was capable of inducing antibacterial activity against a range of MDR strains of Gram-positive and Gram-negative bacteria [50]. Furthermore, a study showed that *D. stramonium* leaf extract composed of 1-Monolinoleoylglycerol trimethylsilyl ether against *E. coli*, *Proteus mirabilis*, *S. aureus, P. aerogenosa* and *K. pneumonia* [51]. Heptasiloxane, hexadecamethyl isolated from *B. cereus* S1 showed potent antibacterial activity against *P*. *florescence, P. aeruginosa*, *S*. *aureus, Vibrio damsela *and *Aeromonas hydrophila* [52]*.* Pu et al. revealed the antibacterial activity of 9, 12-Octadecadienoic acid against *S. aureus, E. coli* and *Salmonella* sp. [53]. A study showed that 9, 12-Octadecadienoic acid present in *Jatropha curcas* synergised with moxifloxacin, ofloxacin, and rifampicin against *A. baumannii, E. coli, E. faecalis, S. aureus,* and *P. aeruginosa* and* P*. *chlororaphis* [51]. Khan et al. showed both antibacterial and clarithromycin synergising potential of oleic acid against *E. coli* and *S. aureus* [54]*.* A study demonstrated the antibacterial potential of cyclohexanecarboxamide against Gram-positive bacteria (*S. aureus*, *S. epidermidis*, *E*. *faecalis*, *Streptococcus pyogenes*, *B. cereus*) and Gram-negative bacteria (*E. coli*, *P. aeruginosa*, *Enterobacter cloacae*, *P*. *vulgaris*) [55]*.* The fruit extract of *Carica papaya,* which comprises 1-Gala-1-ido-octose showed antibacterial activity against *S. aureus, E. coli* and *Salmonella* spp. [56, 57]. Meanwhile, a study showed that 3-Pyridinecarboxylic acid synergised streptomycin against both Gram-negative and Gram-positive bacteria [58]. Lauric acid was a saturated fatty acid that has broad-spectrum antibacterial activity against *Bacteroides* spp. and *Clostridium* spp. also acted synergistically with gentamicin and streptomycin against *S. aureus* [59]. Acetic acid is a carboxylic acid with antibacterial potential against a range of MDR strains of bacteria, can also exhibit synergistic action with vancomycin against *Salmonella* [60, 61]*.* Martins et al. showed the antibacterial potential of Heptacosane against *Prevotella nigrescens* [62]. Another study showed that heptacosane present *in L. nobilis* and apricot *P. armeniaca* essential oils synergised ciprofloxacin and vancomycin against *M. luteus, S. aureus, B. subtilis, E. coli, P. aeruginosa* and *K. pneumonia* [63]. Octadecane 3-ethyl-5-(2-ethylbutyl)- has also been considered as an effective antimicrobial agent, which is present in plants like *Enhalus acoroides* and *Acacia ehrenbergiana* [64, 65]. Asarone was a phenylpropanoid, which was a naturally occurring compound in plants, which was known to have antimicrobial properties [66]. A recent study conducted by Khoa et al. showed that isomers of digoxigenin, such as periplogenin can exist in plants of *Phyllanthaceae* family [67]. An investigation revealed that digoxigenin is a steroid compound capable of potentiating ampicillin against *E. coli* and *S. aureus* [68]. The antibacterial activity of stearic acid, a saturated fatty acid isolated from *Stemodia foliosa* has been observed against *B. cereus, B. subtilis* and *Mycobacterium fortuitum* [69].

### FE-SEM analysis

**Fig. 9:**
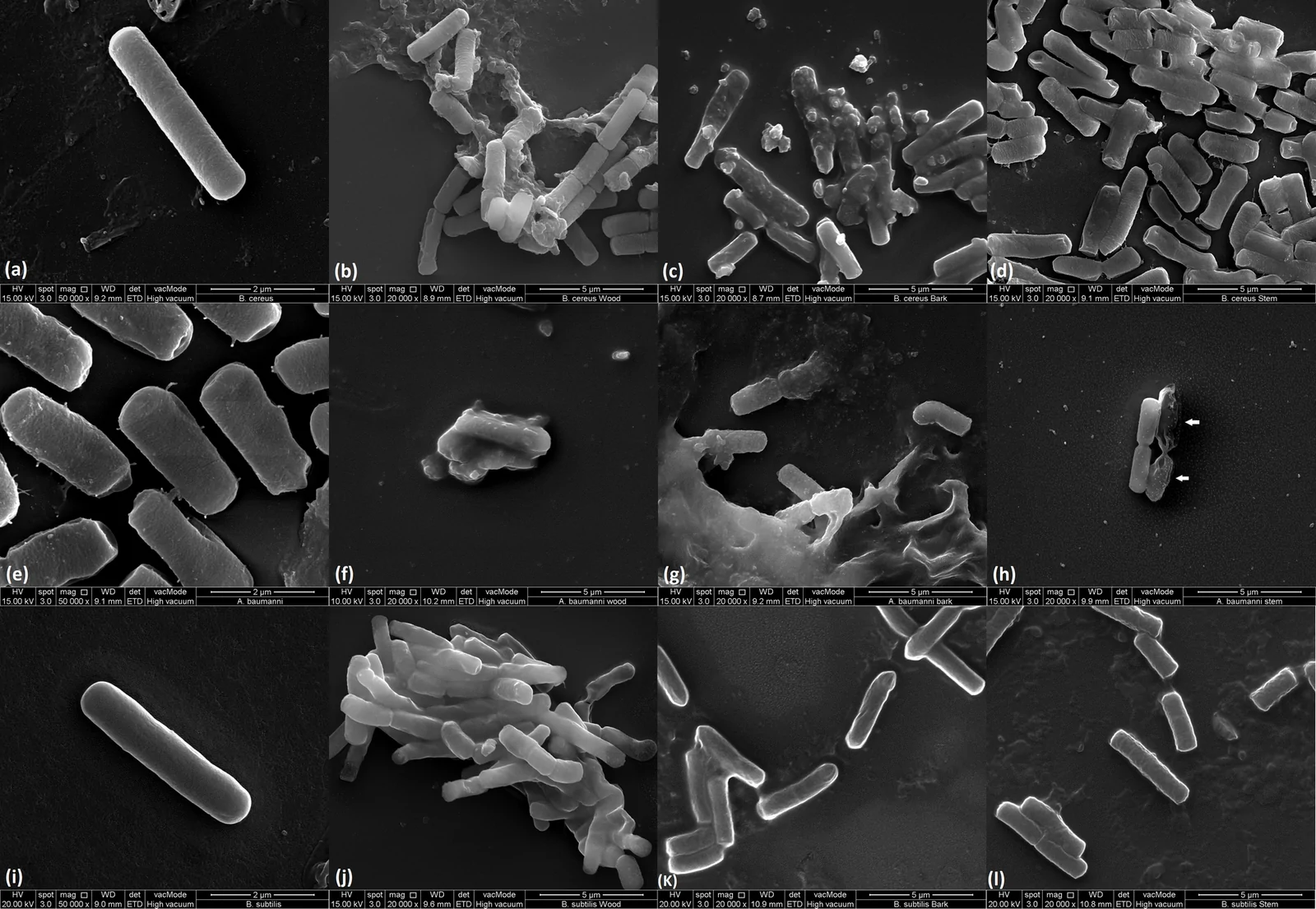
FE-SEM images elucidating morphological changes between untreated bacterial cells (control samples) and plant extract treated bacterial cells taken from the zone of inhibition after 24 h of incubation. (**a**) untreated *B. cereus*. (**b**) *B. cereus* treated with *C. bracteosus* chloroform wood. (**c**) *B. cereus* treated *C. bracteosus* chloroform bark. (**d**) *B. cereus* treated *C. bracteosus* chloroform stem. (**e**) untreated *A. baumannii*. (**f**) *A. baumannii* treated with *C. bracteosus* chloroform wood. (**g**) *A. baumannii* treated *C. bracteosus* chloroform bark. (**h**) *A. baumannii* treated *C. bracteosus* chloroform stem. (**i**) untreated *B. subtilis.* (**j**) *B. subtilis* treated with *C. bracteosus* chloroform wood, (**k**) *B. subtilis* treated with *C. bracteosus* chloroform bark, (**l**) *B. subtilis* treated with *C. bracteosus* chloroform stem

Changes in surface structure of *B. cereus*, *B. subtilis* and *A. baumannii* cells resulting from treatment with each plant extract were analysed by FE-SEM. Non-treated cells remained intact, smooth, and displayed fine structure with uniform cytoplasmic density and cell wall and membrane integrity (Fig. 9a, e and i). In contrast, cells treated with plant extracts displayed clear surface structure trauma, including the appearance of membrane holes, formation of cellular cavities, disappearance of cellular contents, wrinkling, shrinking with alteration of overall cell size, cellular telescoping, invagination, and roughness portraying compromisation of cell membranes with surface protrusions (Fig. 9b-d, f–h and j). Hence, these adverse characteristics indicated that cell lysis had occurred. At a microscopic level, *C. bracteosus* chloroform bark extract appears to have induced a greater degree of damage to the cellular structural integrity of the three selected bacteria compared to other extracts of the plant.

Microscopic techniques have been employed to assess changes in cells for many years. The alterations in the bacterial morphology were determined by scanning electron microscopy (SEM). The appearance of certain cells packed into degenerating bodies with chromatin condensation are considered as hallmarks of cellular trauma caused by antimicrobial agents [70]. It can be inferred that a magnitude of bacterial cells treated by plant extracts may be undergoing microscopically notable degeneration. Therefore, the antimicrobial action mechanisms of the plant extracts may result in bacterial cellular disintegration directly or indirectly [71]. In one study, SEM images of *B*. *cereus* cells treated with the ethanolic extract of *Annona squamosa* showed abnormal elongations, distortions and alterations in bacterial cell shape, depletion of the cytoplasmic mass and leakage of intracellular contents [72]. Whereas Alejandro et al. showed that methanolic extract of *Nothoscordum bivalve* can cause traumatisation of *A. baumannii* cell membrane and biofilm instability under SEM observations [73].

## Conclusion

The present finding of this study shows that extracts of *C. bracteosus* have mild yet broad-spectrum activity against both Gram-positive and Gram-negative bacteria, thus qualifying as a suitable candidate to be recognised as an antibacterial agent with compounds having potent synergistic ability when used in combination with standard antibiotic agents against drug-resistant bacteria. The stem extract appears to be more potent in terms of antibacterial activity than other extracts of the plant. Also, it can be assumed that chloroform extracts of the plant, particularly stem, wood and bark extracts show bacteriostatic activity rather than being bactericidal. Further studies are required to be carried out to broaden the findings of the current research by testing different extracts of the plant against a greater variety of bacterial strains. Although pioneering, the current study poses limitations. Hence, the antibacterial and antibiotic synergistic potentials of extract fractions and isolated compounds of *C. bracteosus* are needed to be determined with greater precision. Future studies are recommended to purify and identify lead phytochemical compounds present in the extracts of *C. bracteosus* using sophisticated techniques such as LCMS and prepHPLC-NMR, and to determine the mechanisms of action of these molecules on inducing antibacterial action and modification of antibiotic molecule binding target sites. Further investigations are necessary to determine the bacterial anti-quorum sensing activity of standalone *C. bracteosus* extracts and *C. bracteosus*-antibiotic combinations. Moreover, studies can be carried out based on the significance of lead molecules using ex vivo and nano-medicine drug delivery applications.

## Supplementary Information

Supplementary Material 1.

Supplementary Material 2.

Supplementary Material 3.

Supplementary Material 4.

## Data Availability

Data is provided within the manuscript or supplementary information files. Further inquiries can be directed to the corresponding authors.

## Abbreviations

°″E: Degrees to East: °″N: Degrees to North
µg: Micrograms
µL: Microliters
ATCC: American Type Culture Collection
*A. baumannii*: *Acinetobacter baumannii*
*A. karoo*: *Acacia karoo*
*B. subtilis*: *Bacillus subtilis*
*B. cereus*: *Bacillus cereus*
*C. bracteosus*: *Cleistanthus bracteosus*
*C. collinus*: *Cleistanthus collinus*
CFU/mL: Colony Forming Units per millilitre
CLSI: Clinical and Laboratory Standards Institute
DMSO: Dimethyl sulfoxide
DNA: Deoxyribonucleic acid
*E. coli*: *Escherichia coli*
FIC: Fractional Inhibitory Concentration
FICI: Fractional Inhibitory Concentration Index
FBC: Fractional Bactericidal Concentration
FBCI: Fractional Bactericidal Concentration Index
OD: Optical density
FRIM: Forest Research Institute of Malaysia
FE-SEM: Field emission scanning electron microscopy
g: Grams
GIIs: Growth inhibitory indices
GPS: Global Positioning System
HPLC: High Performance Liquid Chromatography
IU: International Unit
*K. pneumoniae*: *Klebsiella pneumoniae*
L: Liters
m: Meters
MBC: Minimum bactericidal concentration
mg: Milligram
mg/mL: Milligrams per millilitre
MHA: Mueller-Hinton agar
MHB: Mueller-Hinton broth
MIC: Minimum inhibitory concentration
mL: Milliliters
mm: Millimeters
MRSA: Methicillin-resistant*Staphylococcus aureus*
MTCC: Microbial Type Culture Collection and Gene Bank
*M. charantia*: *Momordica charantia*
n: Sample number
NC: Negative Control
NCIM: National Collection of Industrial Microorganisms
nm: Nanometers
ºC: Celsius degrees
*P. aeruginosa*: *Pseudomonus aeruginosa*
PBP: Penicillin-Binding Protein
PC: Positive Control
RNA: Ribonucleic acid
*S. aureus*: *Staphylococcus aureus*
*S. typhi*: *Salmonella typhi*
SARS-CoV: Severe Acute Respiratory Syndrome-related Coronavirus
S.E.M: Standard error of the mean
SEM: scanning electron microscopy
spp.: Species
UV: Ultraviolet
w/v: Weight to volume ratio
WHO: World Health Organization
β: Beta
mL/min: Milliliters per minute
μg/mL: Micrograms per millilitre
>: Greater than
GC-MS: Gas chromatography–mass spectrometry
LC-MS: Liquid chromatography–mass spectrometry
NMR: Nuclear magnetic resonance
IZD: Inhibition zone diameter
Rf: Retention factor
RRT: Relative retention time
M/Z: Mass-to-charge ratio
VRE: Vancomycin-resistant Enterococcus
PTFE: Polytetrafluoroethylene
