# Supplementary material for: Chemical profiling and evaluation of in vitro antibacterial and antibiotic synergistic activities of Cleistanthus bracteosus Jabl. from Malaysian rainforests
Source: BMC Complement Med Ther. 2026 Jun 20;26:263. doi: 10.1186/s12906-026-05439-7 (PMC13543593; doi:10.1186/s12906-026-05439-7)
Supplement: Supplementary file 1 [file 12906_2026_5439_MOESM1_ESM.docx]

Table S1. The raw masses and the final yield of *C. bracteosus* methanol, *n*-hexane and chloroform extracts.

| Extract type | Extract | Mass of initial plant material (g) | Mass of extracted plant material (g) | Yield of plant extract (%) |
| --- | --- | --- | --- | --- |
| Methanol | Leaves | 70 | 0.534 | 0.762 |
|  | Bark | 70 | 0.511 | 0.73 |
| *n*-Hexane | Leaves | 70 | 0.531 | 0.75 |
|  | Bark | 70 | 0.508 | 0.725 |
| Chloroform | Leaves | 70 | 0.533 | 0.761 |
|  | Bark | 70 | 0.510 | 0.728 |
|  | Stem | 70 | 0.498 | 0.711 |
|  | Wood | 70 | 0.490 | 0.70 |

Table S2. Diameters for the zone of inhibition for methanol extracts of *C. bracteosus* against bacteria in the disc diffusion antibacterial assay.

| Bacterial isolates | Zone of inhibition (mm) | | | |
| --- | --- | --- | --- | --- |
|  | Leaves extract | Bark extract | Gentamicin 10 µg (PC) | Pure DMSO (NC) |
| *E. coli* ATCC 10536 | 0 | 0 | 22 ± 0.33 | 0 |
| *B. subtilis* ATCC 6633 | 7 ± 0.71 | 0 | 23.5 ± 0.25 | 0 |
| *S. aureus* ATCC 11632 | 0 | 0 | 24 ± 0.56 | 0 |
| *B. cereus* ATCC 10876 | 7 ± 0.70 | 0 | 22.5 ± 0.59 | 0 |
| *P. aeruginosa* ATCC 10145 | 0 | 0 | 12 ± 0.66 | 0 |
| *S. typhi* ATCC 6539 | 0 | 0 | 18 ± 0.54 | 0 |
| *E. coli* ATCC 8739 | 0 | 0 | 23 ± 0.13 | 0 |
| *A. baumannii* ATCC 19606 | 7 ± 0.77 | 0 | 23 ± 0.78 | 0 |

PC = positive control, NC = negative control, Extract concentration is at 1000 µg/disc, Values are represented as the mean of triplicates (n=3) ± S.E.M.

Table S3. Diameters for the zone of inhibition for *n*-hexane extracts of *C. bracteosus* against bacteria in the disc diffusion antibacterial assay.

| Bacterial isolates | Zone of inhibition (mm) | | | |
| --- | --- | --- | --- | --- |
|  | Leaves extract | Bark extract | Gentamicin 10 µg (PC) | Pure DMSO (NC) |
| *E. coli* ATCC 10536 | 0 | 0 | 21 ± 0.34 | 0 |
| *B. subtilis* ATCC 6633 | 6.1 ± 0.71 | 0 | 22.5 ± 0.25 | 0 |
| *S. aureus* ATCC 11632 | 0 | 0 | 25 ± 0.56 | 0 |
| *B. cereus* ATCC 10876 | 0 | 7 ± 0.29 | 23.5 ± 0.27 | 0 |
| *P. aeruginosa* ATCC 10145 | 0 | 0 | 19 ± 0.56 | 0 |
| *S. typhi* ATCC 6539 | 0 | 0 | 20 ± 0.58 | 0 |
| *E. coli* ATCC 8739 | 0 | 0 | 26 ± 0.60 | 0 |
| *A. baumannii* ATCC 19606 | 0 | 8 ± 0.45 | 22 ± 0.65 | 0 |

PC = positive control, NC = negative control, Extract concentration is at 1000 µg/disc, Values are represented as the mean of triplicates (n=3) ± S.E.M.

Table S4**.** Diameters for the zone of inhibition for methanol extracts of *C. bracteosus* in the disc diffusion synergistic antibacterial assay.

| Bacterial isolates | Zone of inhibition (mm) | | | | GIIs | |
| --- | --- | --- | --- | --- | --- | --- |
|  | Standard antibiotic disc  with potency | Standard antibiotic disc | Standard antibiotic and extract combination disc | |  | |
|  |  |  | Leaves | Bark | Leaves | Bark |
| *E. coli* ATCC 10536 | Levofloxacin 5 µg | 32 ± 0.58 | 31 ± 0. 59 | 28 ± 0.88 | - | - |
|  | Ciprofloxacin 5 µg | 38 ± 0.56 | 35 ± 0. 69 | 35 ± 0.58 | - | - |
|  | Penicillin G 10 IU (6 µg) | 0 | 0 | 0 | - | - |
|  | Gentamicin 10 µg | 20 ± 0.80 | 17 ± 0.77 | 15 ± 0.40 | - | - |
|  | Chloramphenicol 30 µg | 28 ± 0.48 | 25.5 ± 0.66 | 27 ± 0.88 | - | - |
|  | Ampicillin 10 µg | 10± 0.59 | 9 ± 0. 67 | 9 ± 0.64 | - | - |
| *E. coli* ATCC 8739 | Levofloxacin 5 µg | 33 ± 0.64 | 32 ± 0.57 | 31 ± 0.89 | - | - |
|  | *****Ciprofloxacin 5 µg | 34 ± 0.22 | *****35 ± 0.16 | 32 ± 0.56 | 1.03 | - |
|  | Penicillin G 10 IU (6 µg) | 0 | 0 | 0 | - | - |
|  | Gentamicin 10 µg | 21.5 ± 0.57 | 16 ± 0.55 | 17 ± 0.78 | - | - |
|  | Chloramphenicol 30 µg | 31 ± 0.58 | 27 ± 0.22 | 26.5 ± 0.11 | - | - |
|  | Ampicillin 10 µg | 15 ± 0.55 | 14 ± 0.45 | 15 ± 0.89 | - | - |
| *S. typhi* ATCC 6539 | Levofloxacin 5 µg | 36 ± 0.67 | 35 ± 0.88 | 35 ± 0.67 | - | - |
|  | Ciprofloxacin 5 µg | 40 ± 0.87 | 38 ± 0.32 | 38.5 ± 0.45 | - | - |
|  | Penicillin G 10 IU (6 µg) | 12 ± 0.57 | 12 ± 0.87 | 11.5 ± 0.16 | - | - |

Table S4 (continued)

| Bacterial isolates | Zone of inhibition (mm) | | | | GIIs | |
| --- | --- | --- | --- | --- | --- | --- |
|  | Standard antibiotic disc  with potency | Standard antibiotic disc | Standard antibiotic and extract combination disc | |  |  |
|  |  |  | Leaves | Bark | Leaves | Bark |
|  | Gentamicin 10 µg | 20 ± 0.58 | 15 ± 0.53 | 15 ± 0.45 | - | - |
|  | Chloramphenicol 30 µg | 30 ± 0.34 | 29 ± 0.46 | 30 ± 0.86 | - | - |
|  | Ampicillin 10 µg | 25 ± 0.73 | 24 ± 0.47 | 23 ± 0.54 | - | - |
| *P. aeruginosa* ATCC 10145 | Levofloxacin 5 µg | 22 ± 0.35 | 20 ± 0.22 | 19 ± 0.57 | - | - |
|  | Ciprofloxacin 5 µg | 31 ± 0.52 | 23 ± 0.56 | 27 ± 0.11 | - | - |
|  | Penicillin G 10 IU (6 µg) | 0 | 0 | 0 | - | - |
|  | Gentamicin 10 µg | 13 ± 0.54 | 11.5 ± 0.66 | 10 ± 0.63 | - | - |
|  | Chloramphenicol 30 µg | 0 | 0 | 0 | - | - |
|  | Ampicillin 10 µg | 0 | 0 | 0 | - | - |
| *B. subtilis* ATCC 6633 | Levofloxacin 5 µg | 28 ± 0.57 | 29 ± 0.64 | 29 ± 0.37 | - | - |
|  | Ciprofloxacin 5 µg | 31 ± 0.13 | 31.5 ± 0.25 | 29 ± 0.43 | - | - |
|  | Penicillin G 10 IU (6 µg) | 0 | 0 | 0 | - | - |
|  | Gentamicin 10 µg | 24.5 ± 0.43 | 18 ± 0.57 | 19.5 ± 0.59 | - | - |
|  | Chloramphenicol 30 µg | 26 ± 0.43 | 26 ± 0.22 | 26 ± 0.21 | - | - |
|  | Ampicillin 10 µg | 0 | 0 | 0 | - | - |
| *S. aureus* ATCC 11632 | Levofloxacin 5 µg | 30 ± 0.23 | 29 ± 0.45 | 31 ± 0.57 | - | - |

Table S4 (continued)

| Bacterial isolates | Zone of inhibition (mm) | | | | GIIs | |
| --- | --- | --- | --- | --- | --- | --- |
|  | Standard antibiotic disc  with potency | Standard antibiotic disc | Standard antibiotic and extract combination disc | |  |  |
|  |  |  | Leaves | Bark | Leaves | Bark |
|  | Ciprofloxacin 5 µg | 29 ± 0.24 | 26 ± 0.66 | 29 ± 0.12 | - | - |
|  | *****Penicillin G 10 IU (6 µg) | 17 ± 0.57 | *****18.5 ± 0.60 | *****18 ± 0.43 | 1.09 | 1.06 |
|  | Gentamicin 10 µg | 25 ± 0.59 | 21 ± 0.16 | 19 ± 0.57 | - | - |
|  | Chloramphenicol 30 µg | 31 ± 0.50 | 28 ± 0.34 | 29 ± 0.58 | - | - |
|  | Ampicillin 10 µg | 23 ± 0.51 | 23 ± 0.57 | 23 ± 0.56 | - | - |
| *B. cereus* ATCC 10876 | Levofloxacin 5 µg | 26 ± 0.56 | 27 ± 0.52 | 26 ± 0.51 | - | - |
|  | Ciprofloxacin 5 µg | 28 ± 0.58 | 26 ± 0.55 | 26 ± 0.57 | - | - |
|  | *****Penicillin G 10 IU (6 µg) | 0 | 0 | *****8.5 ± 0.53 | - | **>1** |
|  | Gentamicin 10 µg | 22 ± 0.59 | 16 ± 0.31 | 13 ± 0.57 | - | - |
|  | Chloramphenicol 30 µg | 22 ± 0.74 | 23 ± 0.46 | 23.5 ± 0.26 | - | - |
|  | *****Ampicillin 10 µg | 0 | 6.1 ± 0.16 | *****6.3 ± 0.44 | - | **>1** |
| *A. baumannii* ATCC 19606 | Levofloxacin 5 µg | 27 ± 0.57 | 26 ± 0.87 | 24 ± 0.28 | - | - |
|  | Ciprofloxacin 5 µg | 29 ± 0.16 | 27 ± 0.19 | 26 ± 0.57 | - | - |
|  | *****Penicillin G 10 IU (6 µg) | 0 | 6.1 ± 0.58 | *****8 ± 0.52 | - | >1 |
|  | Gentamicin 10 µg | 22 ± 0.45 | 15 ± 0.57 | 17 ± 0.59 | - | - |
|  | Chloramphenicol 30 µg | 22 ± 0.55 | 22 ± 0.23 | 23 ± 0.97 | - | - |

Table S4 (continued)

| Bacterial isolates | Zone of inhibition (mm) | | | | GIIs | |
| --- | --- | --- | --- | --- | --- | --- |
|  | Standard antibiotic disc  with potency | Standard antibiotic disc | Standard antibiotic and extract combination disc | |  |  |
|  |  |  | Leaves | Bark | Leaves | Bark |
|  | *****Ampicillin 10 µg | 0 | *****7.5 ± 0.54 | *****7.5 ± 0.57 | 1.07 | >1 |

Extract concentration is at 1000 µg/disc, Values for the zone of inhibition are represented as the mean of triplicates (n=3) ± S.E.M, GIIs = Growth inhibitory indices (synergy index). *****Indicates data for antibiotic synergism.

Table S5. Diameters for the zone of inhibition for *n*-hexane extracts of *C. bracteosus* in the disc diffusion synergistic antibacterial assay.

| Bacterial isolates | Zone of inhibition (mm) | | | | GIIs | |
| --- | --- | --- | --- | --- | --- | --- |
|  | Standard antibiotic disc  With potency | Standard antibiotic disc | Standard antibiotic and extract combination disc | |  | |
|  |  |  | Leaves | Bark | Leaves | Bark |
| *E. coli* ATCC 10536 | Levofloxacin 5 µg | 31 ± 0.56 | 30 ± 0. 57 | 29± 0.88 | - | - |
|  | Ciprofloxacin 5 µg | 36 ± 0.53 | 33 ± 0. 67 | 34 ± 0.56 | - | - |
|  | Penicillin G 10 IU (6 µg) | 0 | 0 | 0 | - | - |
|  | Gentamicin 10 µg | 20.5 ± 0.87 | 18 ± 0.78 | 15 ± 0.46 | - | - |
|  | Chloramphenicol 30 µg | 28 ± 0.47 | 26.5 ± 0.69 | 27 ± 0.87 | - | - |
|  | *****Ampicillin 10 µg | 10 ± 0.57 | *****12 ± 0. 66 | *****11 ± 0.64 | 1.2 | 1.1 |
| *E. coli* ATCC 8739 | Levofloxacin 5 µg | 36 ± 0.59 | 36 ± 0.51 | 36 ± 0.57 | - | - |
|  | *****Ciprofloxacin 5 µg | 40 ± 0.57 | *****43.5 ± 0.53 | *****43.5 ± 0.51 | 1.09 | 1.09 |
|  | Penicillin G 10 IU (6 µg) | 0 | 0 | 0 | - | - |
|  | Gentamicin 10 µg | 25 ± 0.54 | 18 ± 0.57 | 22 ± 0.78 | - | - |
|  | Chloramphenicol 30 µg | 34 ± 0.34 | 33 ± 0.23 | 33.5 ± 0.54 | - | - |
|  | Ampicillin 10 µg | 15 ± 0.56 | 14 ± 0.32 | 15 ± 0.45 | - | - |
| *S. typhi* ATCC 6539 | Levofloxacin 5 µg | 36 ± 0.57 | 35 ± 0.37 | 35 ± 0.57 | - | - |
|  | Ciprofloxacin 5 µg | 39 ± 0.76 | 31.5 ± 0.88 | 34 ± 0.34 | - | - |
|  | Penicillin G 10 IU (6 µg) | 13 ± 0.57 | 12.5 ± 0.37 | 12 ± 0.89 | - | - |

Table S5 (continued)

| Bacterial isolates | Zone of inhibition (mm) | | | | GIIs | |
| --- | --- | --- | --- | --- | --- | --- |
|  | Standard antibiotic disc  with potency | Standard antibiotic disc | Standard antibiotic and extract combination disc | |  | |
|  |  |  | Leaves | Bark | Leaves | Bark |
|  | Gentamicin 10 µg | 18 ± 0.55 | 16 ± 0.52 | 18 ± 0.57 | - | - |
|  | Chloramphenicol 30 µg | 31 ± 0.84 | 26 ± 0.45 | 29 ± 0.65 | - | - |
|  | *****Ampicillin 10 µg | 30 ± 0.57 | *****32 ± 0.12 | *****32 ± 0.54 | 1.07 | 1.07 |
| *P. aeruginosa* ATCC 10145 | Levofloxacin 5 µg | 23 ± 0.65 | 19 ± 0.57 | 22.5 ± 0.45 | - | - |
|  | Ciprofloxacin 5 µg | 31.5 ± 0.45 | 28 ± 0.29 | 30 ± 0.56 | - | - |
|  | Penicillin G 10 IU (6 µg) | 0 | 0 | 0 | - | - |
|  | Gentamicin 10 µg | 19.5 ± 0.57 | 7.5 ± 0.56 | 10 ± 0.22 | - | - |
|  | Chloramphenicol 30 µg | 0 | 0 | 0 | - | - |
|  | Ampicillin 10 µg | 0 | 0 | 0 | - | - |
| *B. subtilis* ATCC 6633 | Levofloxacin 5 µg | 26 ± 0.66 | 26 ± 0.34 | 26 ± 0.14 | - | - |
|  | Ciprofloxacin 5 µg | 28 ± 0.57 | 24 ± 0.54 | 27 ± 0.87 | - | - |
|  | Penicillin G 10 IU (6 µg) | 0 | 0 | 0 | - | - |
|  | Gentamicin 10 µg | 21 ± 0.38 | 16 ± 0.26 | 19 ± 0.65 | - | - |
|  | Chloramphenicol 30 µg | 19.5 ± 0.22 | 19 ± 0.65 | 22 ± 0.25 | - | - |
|  | Ampicillin 10 µg | 0 | 0 | 0 | - | - |
| *S. aureus* ATCC 11632 | Levofloxacin 5 µg | 31 ± 0.64 | 29 ± 0.27 | 30.5 ± 0.93 | - | - |

Table S5 (continued)

| Bacterial isolates | Zone of inhibition (mm) | | | | GIIs | |
| --- | --- | --- | --- | --- | --- | --- |
|  | Standard antibiotic disc  with potency | Standard antibiotic disc | Standard antibiotic and extract combination disc | |  | |
|  |  |  | Leaves | Bark | Leaves | Bark |
|  | Ciprofloxacin 5 µg | 29 ± 0.48 | 25 ± 0.57 | 29 ± 0.54 | - | - |
|  | *****Penicillin G 10 IU (6 µg) | 14 ± 0.57 | *****16.5 ± 0.18 | *****16.5 ± 0.48 | 1.18 | 1.18 |
|  | Gentamicin 10 µg | 24 ± 0.87 | 20 ± 0.35 | 18.5 ± 0.27 | - | - |
|  | Chloramphenicol 30 µg | 31 ± 0.65 | 29 ± 0.38 | 28 ± 0.35 | - | - |
|  | Ampicillin 10 µg | 41 ± 0.53 | 40 ± 0.28 | 41 ± 0.29 | - | - |
| *B. cereus* ATCC 10876 | Levofloxacin 5 µg | 30 ± 0.57 | 26 ± 0.54 | 27 ± 0.53 | - | - |
|  | *****Ciprofloxacin 5 µg | 30 ± 0.59 | *****32 ± 0.66 | 31 ± 0.94 | 1.07 | - |
|  | Penicillin G 10 IU (6 µg) | 0 | 0 | 0 | - | - |
|  | Gentamicin 10 µg | 19 ± 0.28 | 14.5 ± 0.28 | 19 ± 0.49 | - | - |
|  | Chloramphenicol 30 µg | 24 ± 0.45 | 24 ± 0.44 | 23 ± 0.57 | - | - |
|  | Ampicillin 10 µg | 0 | 0 | 0 | - | - |
| *A. baumannii* ATCC 19606 | Levofloxacin 5 µg | 25 ± 0.65 | 25 ± 0.34 | 27 ± 0.23 | - | - |
|  | Ciprofloxacin 5 µg | 26 ± 0.96 | 24 ± 0.54 | 30 ± 0.57 | - | - |
|  | Penicillin G 10 IU (6 µg) | 0 | 0 | 0 | - | - |
|  | Gentamicin 10 µg | 20.5 ± 0.39 | 19.5 ± 0.20 | 22 ± 0.10 | - | - |
|  | Chloramphenicol 30 µg | 20 ± 0.57 | 20 ± 0.56 | 18 ± 0.84 | - | - |

Table S5 (continued)

| Bacterial isolates | Zone of inhibition (mm) | | | | GIIs | |
| --- | --- | --- | --- | --- | --- | --- |
|  | Standard antibiotic disc  with potency | Standard antibiotic disc | Standard antibiotic and extract combination disc | |  | |
|  |  |  | Leaves | Bark | Leaves | Bark |
|  | Ampicillin 10 µg | 0 | 0 | 0 | - | - |

Extract concentration is at 1000 µg/disc, Values for the zone of inhibition are represented as the mean of triplicates (n=3) ± S.E.M, GIIs = Growth inhibitory indices (synergy index). *****Indicates data for antibiotic synergism.

Table S6. Indicates a summary of results obtained for the qualitatively analysed phytochemical profile of *C. bracteosus* extracts.

| Phytochemical compound | Chloroform | | | | Methanol | | *n*-Hexane | | Observation |
| --- | --- | --- | --- | --- | --- | --- | --- | --- | --- |
|  | Bark | Leaves | Stem | Wood | Bark | Leaves | Bark | Leaves |  |
| Flavonoids | - | + | + | + | - | - | + | - | Intense yellow colouration of chloroform leaves and *n-*hexane bark extracts |
| Alkaloids | + | - | + | + | - | - | - | - | Extracts turns orange to red in colour |
| Phenols | - | - | - | - | + | + | - | - | Dark bluish green black colouration of methanol leaves and bark extracts |
| Coumarins | - | + | + | + | - | - | + | - | Intense yellow colouration of chloroform leaves and *n*-hexane bark extracts |
| Saponins | + | + | + | + | - | - | - | - | A thick foam of nearly 3 cm in height was produced |
| Tannins | - | - | - | - | + | + | - | - | Dark bluish green black colouration of methanol leaves and bark extracts |

The sign (+) indicates presence and the sign (-) indicates absence.

Table S7. TLC compound separations for chloroform bark extract of *C. bracteosus* under 70 % *n*-hexane and 30 % ethyl acetate.

| Fraction number | Rf value | | | | |  |
| --- | --- | --- | --- | --- | --- | --- |
|  | Visible light (Normal) | UV 254 | UV 356 | Dragendorff's reagent | Vanilin + Sulfuric acid | 10 % Sodium hydroxide |
| 1 | 0 | - | - | 0 | - | - |
| 2 | 0.46 | - | - | - | - | 0.46 |
| 3 | 0.6 | - | - | - | 0.6 | - |
| 4 | 0.7 | - |  | - | - | - |
| 5 | 0.77 |  |  | - | - | - |
| 6 | - |  | 0.8 | - | - | - |
| 7 | - | 0.83 |  | - | - | - |

Table S8. TLC compound separations for chloroform stem extract of *C. bracteosus* under 70 % *n*-hexane and 30 % ethyl acetate.

| Fraction number | Rf value | | | | |
| --- | --- | --- | --- | --- | --- |
|  | Visible light (Normal) | UV 254 | UV 356 | Vanilin + Sulfuric acid | 10 % Sodium hydroxide |
| 1 | 0.26 | - | - | - | - |
| 2 | 0.4 | - | - | 0.4 | 0.4 |
| 3 | 0.53 | - | - | - | - |
| 4 | 0.6 | - | - | - | 0.6 |
| 5 | 0.63 | - | - | - | 0.63 |
| 6 | 0.7 | - | - | - | - |
| 7 | 0.8 | - | - | 0.7 | - |
| 8 | - | - | 0.83 | - | - |
|  |  |  |  |  |  |

Table S9. TLC compound separations for chloroform wood extract of C. bracteosus under 70 % *n*-hexane and 30 % ethyl acetate.

| Fraction number |  | Rf value | | | | | |
| --- | --- | --- | --- | --- | --- | --- | --- |
|  | Visible light (Normal) | UV 254 | UV 356 | Dragendorff's reagent | Vanilin + Sulfuric acid | 10 % Sodium hydroxide | Ferric chloride |
| 1 | 0 | - | - | 0 | - | - | - |
| 2 | 0.23 | - | - | - | 0.23 | 0.23 | - |
| 3 | 0.63 | - | - | - | - | 0.63 | - |
| 4 | 0.77 | - | - | - | - | - | - |
| 5 | - | - | 0.8 | - | 0.8 | - | - |
| 6 | - | 0.87 | - | - | 0.87 | - | - |
| 7 | - | - | - | - | - | - | 0.33 |

Table S10. Phytochemical compounds detected in chloroform stem, bark and wood fractions of *C. bracteosus* in GC-MS analysis.

|  | | |  | GC-MS | | | HPLC |
| --- | --- | --- | --- | --- | --- | --- | --- |
| Type | Extract fraction | Positive bioactive property of fraction | Compound detected | M/Z | RRT | Area % | RT |
| Stem | F1 | Synergistic with ampicillin and penicillin G | Hexadecanoic acid, methyl ester | 270 | 8.82 | 2.557 | - |
|  |  |  | Methyl stearate | 298 | 11.25 | 4.005 | - |
|  |  |  | Octadecanamide | 283 | 13.19 | 1.779 | - |
|  | F2 | Synergistic with ampicillin and penicillin G | 4-Methyl-2,4-bis(4'-trimethylsilyloxyphenyl) pentene-1 | 412 | 15.79 | 0.211 | - |
|  |  |  | Trimethyl [4-(1,1,3,3,-tetramethylbutyl) phenoxy] silane | 278 | 18.69 | 0.351 | - |
|  | F3 | Synergistic with penicillin G | Benzenedodecanoic acid | 378 | 23.09 | 0.658 | - |
|  |  |  | Hexadecanoic acid, methyl ester | 270 | 21.68 | 0.864 | - |
|  | F4 | Synergistic with ampicillin and penicillin G | Hexasiloxane | 431 | 6.92 | 2.973 | - |
|  |  |  | Octasiloxane | 563 | 10.46 | 1.031 | - |
|  |  |  | 1-Monolinoleoylglycerol trimethylsilyl ether | 469 | 16.62 | 1.944 | - |
|  |  |  | Heptasiloxane, hexadecamethyl | 517 | 8.29 | 1.693 | - |
|  | F5 | Standalone antibacterial activity and synergistic with penicillin G | 9, 12-Octadecadienoic acid | 280 | 22.93 | 0.301 | - |
|  |  |  | 1-Monolinoleoylglycerol trimethylsilyl ether | 469 | 17.24 | 0.595 | - |
|  |  |  | Oleic acid | 282 | 17.4 | 1.320 | - |
|  | F6 | Standalone antibacterial activity and synergistic with penicillin G | Hexadecanoic acid, methyl ester | 270 | 22.61 | 1.250 | - |
|  |  |  | 1-Monolinoleoylglycerol trimethylsilyl ether | 469 | 17.04 | 1.396 | - |
|  | F7 | Standalone antibacterial activity and synergistic with penicillin G | Hexadecanoic acid, methyl ester | 270 | 22.94 | 2.608 | - |
|  |  |  | 9,12,15-Octadecatrienoic acid | 443 | 22.92 | 2.608 | - |
|  | F8 | Standalone antibacterial activity and synergistic with penicillin G | 7aH-Cyclopenta(a)cyclopropa(f)cycloundecene-2,4,7,7a,10,11-hexol,1,1a,2,3,4,4a | 580 | 14.42 | 0.883 | - |
|  |  |  | Hexadecanoic acid, methyl ester | 270 | 14.44 | 0.587 | - |
|  | F9 | Synergistic with penicillin G | Hexadecanoic acid, methyl ester | 270 | 16.25 | 1.973 | - |
| Bark | F1  F2 | Standalone antibacterial activity  Standalone antibacterial activity | 4-Methyl-2,4-bis(4'-trimethylsilyloxyphenyl) pentene-1 | 412 | 18.87 | 1.212 | - |
|  |  |  | Oleic acid | 282 | 12.44 | 0.815 | - |
|  | F3  F4 | Standalone antibacterial activity  Synergistic with ampicillin and penicillin G | Oleic acid | 282 | 23.04 | 0.638 | - |
|  |  |  | Acevaltrate | 480 | 16.03 | 0.536 | - |
|  |  |  | α-Asarone | 209 | 17.70 | 0.354 | - |
|  |  |  | 1-Monolinoleoylglycerol trimethylsilyl ether | 469 | 13.68 | 0.705 | - |
|  | F5 | Standalone antibacterial activity | 1-Gala-1-ido-octose | 240 | 14.46 | 0.541 | - |
|  | F6 | Standalone antibacterial activity and synergistic with ampicillin and penicillin G | Digoxigenin | 388 | 2.68 | 4.176 | 4.512 |
|  |  |  | Lauric acid or dodecanoic acid | 200 | 9.32 | 0.769 | 6.772 |
|  | F7 | - | Lauric acid or dodecanoic acid | 200 | 16.26 | 2.167 | - |
| Wood | F1 | - | β-D-Glucopyranosiduronic acid | 228 | 22 | 0.558 | - |
|  | F2 | - | Tetradecanoic acid | 326 | 18.96 | 0.691 | - |
|  | F3 | - | Gibberellic acid | 346 | 2.81 | 0.679 | - |
|  |  |  | Acetic acid | 60 | 7.6 | 0.322 | - |
|  | F4 | Standalone antibacterial activity and synergistic with penicillin G | Stearic acid | 594 | 18.07 | 0.365 | - |
|  | F5 | - | 4-Methyl-2,4-bis(4'-trimethylsilyloxyphenyl) pentene-1 | 412 | 13.19 | 0.263 | - |
|  |  |  | Trimethyl [4-(1,1,3,3,-tetramethylbutyl) phenoxy] silane | 278 | 22.03 | 0.207 | - |
|  | F6 | - | 9, 12-Octadecadienoic acid | 443 | 20.68 | 0.486 | - |
|  | F7 | - | Heptacosane | 380 | 13.62 | 0.088 | - |
|  |  |  | Octadecane 3-ethyl-5-(2-ethylbutyl)- | 366 | 15.31 | 2.277 | - |

F = Isolated fraction of extract, M/Z = Mass-to-charge ratio, RRT = Relative retention time, RT = Retention time.

Supplementary Figure 1. Presentation of antibacterial assay analysed data using GraphPad Prism.

Supplementary Figure 2. GC-MS chromatograms of *C. bracteosus* chloroform (a) bark, (b) stem and (c) wood extract fractions. X-axis indicates relative retention time (RRT) and Y-axis indicates area percentage (%).

Supplementary Figure 3. HPLC chromatogram of TLC isolated fraction number 6 of *C. bracteosus* chloroform bark extract. Digoxigenin at RT 4.512 mins (a). Digoxigenin reference HPLC signal [28] (b). Lauric acid at RT 6.772 mins (c). Lauric acid reference HPLC signal [29] (d). HPLC signal of DCM as standard (e).
